# Supplementary material for: Assessing coupling interactions in a safe and just operating space for regional sustainability
Source: Nat Commun. 2023 Mar 13;14:1369. doi: 10.1038/s41467-023-37073-z (PMC10011604; doi:10.1038/s41467-023-37073-z)
Supplement: Supplementary file 1 — Supplementary Information [file 41467_2023_37073_MOESM1_ESM.pdf]

Supplementary Information for

Assessing coupling interactions in a safe and just operating space for regional sustainability

Dongni Han[1], Deyong Yu[1,2,3*], Jiangxiao Qiu[4]

[1]State Key Laboratory of Earth Surface Processes and Resource Ecology (ESPRE), Faculty of Geographical Science, Beijing Normal University, Beijing 100875, China

[2]Key Laboratory of Tibetan Plateau Land Surface Processes and Ecological Conservation, Qinghai Normal University, Xining 810016, China

[3]Academy of Plateau Science and Sustainability, People's Government of Qinghai Province and Beijing Normal University, Xining 810016, China

[4]School of Forest, Fisheries, and Geomatics Sciences, Fort Lauderdale Research and Education Center, University of Florida, Davie, FL, USA.

Supplementary information:

Supplementary information comprises two main sections. The first, "Methodology and data", describes: (i) methodology flow chart; (ii) environmental limits and footprints; (iii) social foundations and human well-being; (iv) coupling coordination degree model; and (v) data sources. The second, "Supplementary results", presents additional findings omitted in the main manuscript due to space limitations.

1 Methodology and data

1.1 Method graphical abstract

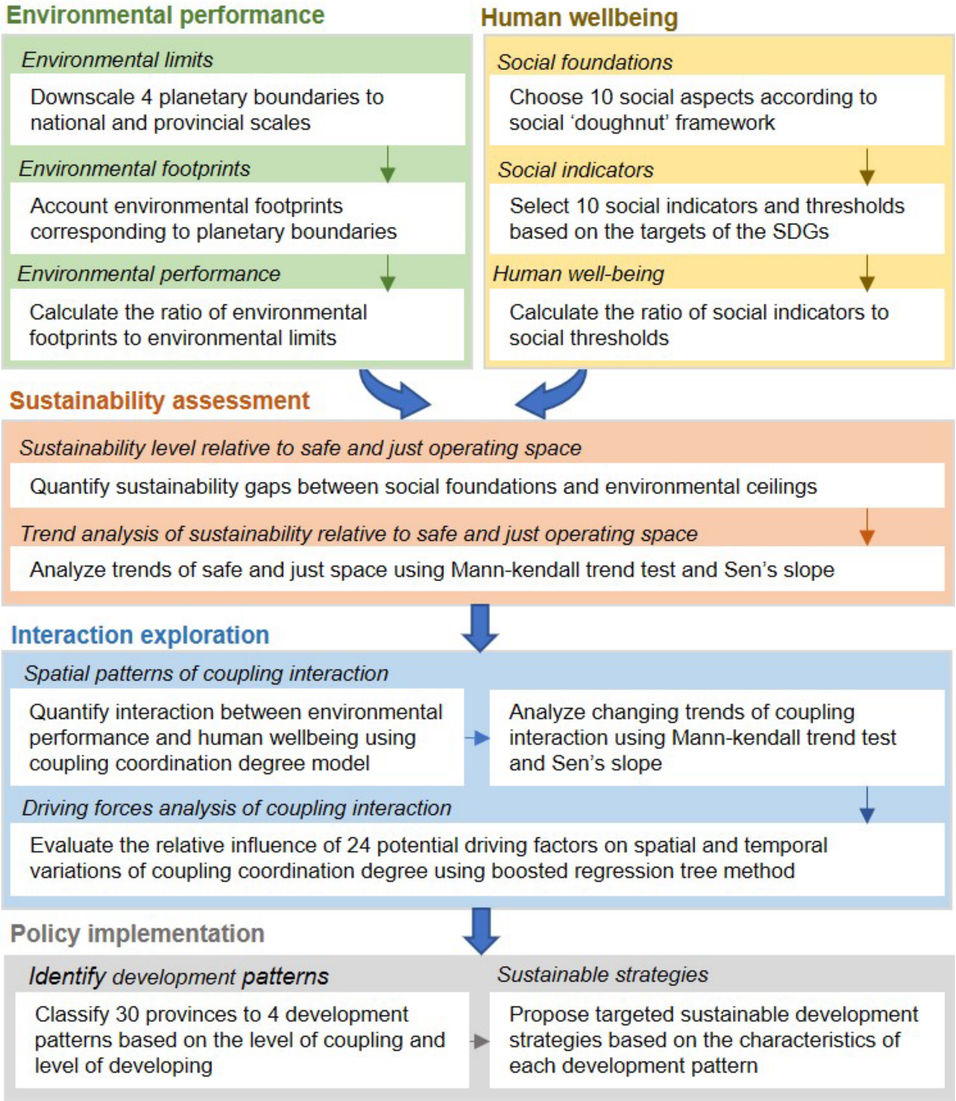

Fig. S1| Overview of methodological steps for assessing coupling relationships within a safe and just operating space framework for sustainability assessment.

1.2 Environmental limits and environmental footprints

Considering the cross-scale interactions of biophysical processes[1]. Steffen et al. (2015)[2] revise the planetary boundaries in a way that some of them with spatial heterogeneity may have regional or local boundaries as complements to their PBs (i.e., biogeochemical flows, freshwater use, and land-system change). These biophysical processes are all found to have threshold behaviors, either with global effects, or with regional or local effects that in aggregate may have global significance[2].

According to Turner II et al. (1990)[3], two types of global environmental changes are differentiated. One type is systemic process. The systemic processes include local sources of changes leading to global effects and global limits. This is the case for climate change. Climate change fits into a systemic process whose explicitly global nature enables this boundary to be one of the few PBs that can be downscaled to the national level through a top-down approach, which appears to be a normative or political issue more than a scientific issue[4]. The other type is aggregated process. The aggregated processes include multiple transformations with local

effects, but they occur worldwide and can have global consequences. This is the case for freshwater use, biogeochemical flows, and land-system change. These PBs are likely to show national threshold behaviors, it is appropriate for measuring the environmental boundaries on a nation-specific basis[4]. By contrast, when it comes to biodiversity loss and chemical pollution, for instance, the high geographical heterogeneity makes it possible to quantify the local boundaries, rather than the nation-wide boundaries[5].

In our analysis, national performance means performance on such parameters that countries and human actors can directly control. One of the main challenges with the PB framework is that it includes a mix of boundaries defined as states of the environment and as pressures driven by human activities. Another challenge is that some boundaries are truly global whereas others are aggregated local or regional processes that result in global effects. In order to discuss national performance, therefore, each needs to be individually reviewed, and a methodology developed that translates the global boundary into a national counterpart while maintaining a clear link to the original problem definition. In consequence, our study proposes quantified national boundaries and indicators for measuring national performance on only four downscaled PBs: climate change, freshwater use, biogeochemical flows, and land-system change. As two indicators are measured for biogeochemical flow boundary (nitrogen and phosphorus cycle), five environmental indicators are considered. For the remaining PBs, it will be significantly more challenging to downscale them meaningfully to the national level.

It has been widely recognized that the equity-based principle should be respected in allocating the planetary boundaries to the national level[5]. Humanity everywhere faces similar threats and the impact of humanity on the Earth are direct and equal[6]. Allocating the planetary boundaries to countries based on a per capita equivalent can normalize inequalities in resource endowment between countries and harmonize the comparative advantage of countries with abundant resources over countries with limited resources, such as land, water resources[7]. This widely employed downscaling technique considers that every human has an equal right to global land resources and allocates environmental limits to countries based on their proportion of the global population[8].

In practice, the equity-based sharing principles can be interpreted in a number of ways, such as equality, sovereignty, right to development, responsibility, capacity, and voluntarism[9]. In the case of the equality principle, national environmental boundaries can be identified by multiplying world-average per capita boundaries with the population of a nation[10].

1.2.1 Climate change

The planetary boundary for climate change has been set as a maximum 350 ppm of atmospheric CO_2 concentration, or 1 W per m^2 of energy imbalance at top of atmosphere[2]. As an alternative boundary, we assume that global warming should be limited to no more than 2 °C above the pre-industrial values, roughly equivalent to 350 ppm[11].

To assess this boundary, the control variable of yearly CO_2 emissions has been selected, since the link between CO_2 emissions and the atmospheric CO_2 concentration is based on strong scientific evidence[2]. Temperature target associated with a global budget can be translated into per capita emissions. About 1900Gt CO_2 had already been emitted by 2011[11]. Remaining cumulative CO_2 emissions for a “medium” probability (50%) to stay below a 2 °C increase from 2011 to 2100 compared with pre-industrial level[11] are approximately 1300Gt CO_2, as shown in Supplementary Table 1. We use the same per capita boundary for both territorial and consumptive performance.

Supplementary Table 1. Cumulative carbon dioxide (CO_2) emissions consistent with limiting warming to less than stated temperature limits at different levels of probability, lines of evidence.

Cumulative CO_2 emissions from 2011 in $GtCO_2$									
Net anthropogenic warming	<1.5°C			<2°C			<3°C		
Fraction of simulations meeting goal	66%	50%	33%	66%	50%	33%	66%	50%	33%
Cumulative emissions	2250	2250	2550	2900	3000	3300	4200	4500	4850
Remaining emissions	400	550	850	1000	1300	1500	2400	2800	3250

Data source: IPCC (2013).

Taking the per capita boundary in 2018 as an example, remaining emissions from 2011 to 2100 are 1300GtCO_2. Total CO_2 emissions from 2011 to 2017 are 253Gt CO_2[12]. Remaining cumulative emissions (carbon budget) for 2018 are equal to 1047 Gt CO_2. The sum of inhabitants from 2018 to 2100 is 814.44 billion people-year[13]. Thus, equal per capita allocations to all inhabitants would be translated to 1.27 tons CO_2 per capita in 2018. The per capital boundaries from 2000 to 2017 are calculated in the same ways. The sum of inhabitants over the years is computed using the United Nations Population Division[13] estimation of the world population until 2050, then assuming medium variant until 2100. Using the calculation process for 2018 as an example, the computation is 7.63 billion in 2018 + 7.71 in 2019 +… + 9.74 in 2050 + … 10.88 in 2100 = 814.44 billion people-year. Downscaled per capita boundaries for climate change are shown in Supplementary Table 2.

Overall, despite the problems with converting the measuring of atmospheric concentration to a measure of annual per capita emissions, this PB is one of the more robust in terms of data availability and the scientific consensus around boundary levels.

Supplementary Table 2. Climate change per capita boundaries from 2000 to 2018.

	Yearly CO_2 emissions /Gton	Cumulative emissions /Gton	Remaining emissions /Gton	World population /billion people-year	Per capita boundary /ton
2000	25.60	325.48	1625.48	936.91	1.73
2001	25.90	299.88	1599.88	930.79	1.72
2002	26.34	273.99	1573.99	924.60	1.70
2003	27.57	247.64	1547.64	918.33	1.69
2004	28.88	220.07	1520.07	911.97	1.67
2005	29.91	191.20	1491.20	905.54	1.65
2006	30.96	161.29	1461.29	899.03	1.63
2007	32.18	130.33	1430.33	892.44	1.60
2008	32.35	98.15	1398.15	885.76	1.58
2009	31.96	65.80	1365.80	879.00	1.55
2010	33.84	33.84	1333.84	872.16	1.53
2011	34.92	0.00	1300.00	865.24	1.50
2012	35.36	34.92	1265.08	858.23	1.47
2013	35.97	70.28	1229.72	851.15	1.44
2014	36.33	106.25	1193.75	843.98	1.41
2015	36.31	142.58	1157.42	836.72	1.38
2016	36.75	178.89	1121.11	829.38	1.35
2017	37.18	215.64	1084.36	821.95	1.32
2018	37.89	252.82	1047.18	814.44	1.29

Data source: IPCC (2013), EDGAR (2019), and UNPD (2019).

To estimate national and provincial territorial performance relative to this per capita boundary, global and national CO_2 emissions are obtained from EDGAR database (https://edgar.jrc.ec.europa.eu/), and provincial CO_2 emissions are obtained from Carbon Emission Accounts & Datasets (CEADs) database (http://www.ceads.net.cn/)[14,15,16]. The CO_2 emissions in this dataset were estimated in terms of the IPCC administrative territorial-based accounting scope. These data represent the CO_2 emissions from both fossil fuel combustion (energy-related emissions) and cement production (process-related emissions) in the emission

accounts. Energy-related CO_2 emissions are converted from the carbon content in fossil fuels, such as raw coal and gasoline during combustion. Emissions are calculated using mass balances according to the IPCC guidelines[7]. The provincial energy inventories are collected from *China Energy Statistical Yearbook 2000-2018*, Department of Energy Statistics of National Bureau of Statistics of the People's Republic of China (China statistics press, 2000-2018). Data for measuring national consumptive performance are obtained from the Eora multi-region input-output (MRIO) database (http://worldmrio.com/). These data represent the consumption-based allocation of CO_2 emissions from energy production (excluding biomass burning) and cement production.

1.2.2 Freshwater use

The original planetary boundary for freshwater use is developed based on the finding that a critical threshold is often crossed if withdrawals of renewable water resources in a watershed exceed 40%[1]. To downscale this boundary, the control variable of the freshwater use has been chosen. The safe amount of freshwater that human can consume globally are 4000 km^3, which corresponds to 40% of total global renewable water resources. Thus, this boundary can be expressed a maximum global withdrawal of 4000 km^3 per year of blue water from rivers, lakes, reservoirs, and renewable groundwater stores. We divide this boundary by world population to arrive at a per capita boundary. We use the same per capita boundary for both territorial and consumptive performance.

To estimate national and provincial territorial performance relative to this per capita boundary, blue water footprint data are obtained from *China Environmental Statistical Yearbook (2000-2018)*. These data refer to the gross amount of water taken by all types of water users, including water transmission losses. Data for consumption-based estimate of national performance are obtained from the Eora multi-region input-output (MRIO) database (http://worldmrio.com/). These data represent the consumption-based allocation of blue water footprint.

The final performance relative to freshwater use boundary is calculated as the ratio of blue water footprints compared to their respective downscaled boundaries.

1.2.3 Land-system change

Originally, the planetary boundary for land-system change is defined as a maximum of 15% of the ice-free land being used for cropland[1]. The global limit is that anthropized surface does not exceed 15% of ice-free land (water bodies excluded). Downscaling this boundary, we choose the surface of anthropized land as the control variable. The surface of anthropized land considered covers agricultural land (arable land and permanent crops) and urbanized land (considered as sealed land). We divide this boundary by global population to arrive at a per capita boundary. We use the same per capita boundary for both territorial and consumptive performance.

To estimate national and provincial territorial performance in relation to this per capita boundary, global and national land footprint data are obtained from the FAOSTAT database (https://www.fao.org/faostat/), and provincial data are obtained from *China Land and Resources Statistical Yearbooks (2000-2018)*. Data for measuring national consumptive performance are obtained from the Eora multi-region input-output (MRIO) database (http://worldmrio.com/). These data represent the consumption-based allocation of cropland area.

The final performance relative to land-system change boundary is calculated as the ratio of land footprints compared to their respective downscaled boundaries.

1.2.4 Biogeochemical flows

The original planetary boundary for phosphorus cycle is 6.2 Tg/year phosphorus mined and applied to erodible (agricultural) soils[2]. To assess this boundary, we choose the control variable of allocation of phosphorus fertilizer applied to cropland. We divide this boundary by the global total population obtained from World Bank (https://data.worldbank.org/) to yield uniform annual boundaries. We use the same per capita boundary for both territorial and consumptive performance. Phosphorus footprints represent the allocation of phosphorus fertilizer applied to cropland. Global and national territorial data are obtained from the FAOSTAT database (https://www.fao.org/faostat/), and provincial data are obtained from *China Rural Statistical Yearbooks (2000-2018).* Data for measuring national consumptive performance are obtained from the Eora multi-region input-output (MRIO) database (http://worldmrio.com/).

The planetary boundary for nitrogen cycle is 62 Tg/year nitrogen, including intended biological and chemical N fixation[2]. The nitrogen footprint does not include all reactive nitrogen, as it does not include biological fixation. According to Steffen et al., (2015)[2], the current value of N flow is 150 Tg N per annum, out of which 96 Tg N per annum (64%) is attributed to chemical fixation by fertilizers. Algunaibet et al., (2019)[17] reduced the N cycle planetary boundary from 62 to 39.7 Tg N per annum to consider industrial fixation only, assuming such share would remain constant. Allocation of nitrogen fertilizer applied to cropland has been selected as the control variable. We divide the planetary boundaries for Nitrogen cycle by the global total population obtained from World Bank (https://data.worldbank.org/) to yield uniform annual boundaries. We use the same per capita boundary for both territorial and consumptive performance. Nitrogen footprints represent the allocation of nitrogen fertilizer applied to cropland. Global and national territorial data are obtained from the FAOSTAT database (https://www.fao.org/faostat/), and provincial data are obtained from *China Rural Statistical Yearbooks (2000-2018).* Data for measuring national consumptive performance are obtained from the Eora multi-region input-output (MRIO) database (http://worldmrio.com/). The final performance relative to biogeochemical flows is calculated as the ratio of nitrogen and phosphorus footprints compared to their respective disaggregated boundaries.

1.2.5 Other planetary boundaries

Two of nine planetary boundaries have not been quantified, i.e., novel entities and atmospheric aerosol loading boundaries. For three of nine PBs, a number of limiting factors mean that it is not currently possible to downscale the planetary-level boundaries to the national level in a meaningful way and stay true to the original methods and boundary definitions.

Biosphere integrity is not explicitly included in the analysis due to the large difficulty in measuring and downscaling both functional and genetic diversity. As the trends are very long term and the range of uncertainty at the global level is measured in orders of magnitude (e.g., the current rate of extinction is estimated to be between 100 and 1000 times higher than pre-Anthropocene).

Ocean acidification is not included as a separate boundary since it is mainly driven by climate change, thus the corresponding pressure indicator (i.e., CO_2 emissions) is already fully accounted for in the analysis. According to Steffen et al. (2015)[2], the ocean acidification boundary would not be transgressed if the climate change boundary of 350 ppm CO_2 concentration were to be respected.

The stratospheric ozone depletion boundary is expressed as a<5% reduction in O_3 concentration from pre-industrial level, which is not included in our analysis. As the minimum O_3 concentration has been steady for about 15 years and is expected to rise over the coming decades as the ozone hole is repaired after the phasing out of ozone-depleting substances. Besides, because of the longevity of ozone depleting substances, however, it is unclear how such a boundary on a pressure should be formulated.

1.2.6 Assessing environmental performance

The quantification of environmental performance is calculated as the ratio of environmental footprint to environmental limits. Environmental performance is classified into three categories, referring to Steffen et al. (2015)[2], shown in Supplementary Table 3.

Supplementary Table 3. Environmental performance defined with three categories.

Performance	Color	Description
Safe	(green)	Below boundary
Increasing risk	(yellow)	In zone of uncertainty
High risk	(red)	Beyond zone of uncertainty

Source: Rockström et al., 2009b[18] and Steffen et al., 2015[2].

A zone of uncertainty is associated with each of the boundaries. This zone contains gaps and weaknesses in the scientific knowledge base and the inherent uncertainties in the functioning of the Earth System. At the "safe" end of the zone of uncertainty, current scientific knowledge suggests that there is very low probability of crossing a critical threshold or substantially eroding the resilience of the Earth System. Beyond the "danger" end of the zone of uncertainty, current knowledge suggests a much higher probability of changes in the functioning of the Earth System that could cause damage to human society. The zones of uncertainty for origin PBs are referred to Steffen et al., (2015)[2]. In addition, we have revised the zone of uncertainty for nitrogen cycle boundary only including intended chemical N fixation based on the research of Algunaibet et al., (2019)[17].

Supplementary Table 4. The control variables, along with the proposed planetary boundaries and zones of uncertainty.

Earth System process	Control variable	Planetary Boundary	Zone of uncertainty
Climate change	Atmospheric CO_2 concentration	350ppm	350-450ppm
Freshwater use	Maximum amount of blue water use	4000 km^3 yr^{-1}	4000–6000 km^3 yr^{-1}
Land-system change	Ice-free land surface converted to cropland	15%	15%– 20%
Nitrogen cycle	Industrial fixation of N (Chemical fertilizer)	39.7 T g N yr^{-1}	39.7–52.5 T g N yr^{-1}
Phosphorus cycle	P flow from fertilizers to erodible soils	6.2 T g P yr^{-1}	6.2-11.2 T g P yr^{-1}

Source: Steffen et al., 2015[2] and Algunaibet et al., 2019[17].

1.3 Social foundations and human well-being

We choose 10 aspects of the social foundation according to the Oxfam[19]. Social indicators and thresholds for each aspect are selected based on the targets of the Sustainable Development Goals (SDGs) of the United Nations.

1.3.1 Food security

The target in the Sustainable Development Goals (SDGs) is to end all forms of malnutrition, including achieving, by 2025, the internationally agreed targets on stunting and wasting in children under 5 years of age, and address the nutritional needs of adolescent girls, pregnant and lactating women and older persons (Goal 2). The SDG indicator is prevalence of undernourishment. We choose a threshold of 95% for this indicator, realizing the difficulty of providing universal access to at least 5% of the population. In our study, percentage of undernourished Children under 5 is selected as our food security indicator. The data for China and the World are from Work Bank (https://data.worldbank.org/). The data for provinces are from *China Health Statistics Yearbooks (2000-2018).*

1.3.2 Income

The target in the Sustainable Development Goals is to eradicate extreme poverty for all people everywhere, currently measured as people living on less than $1.25 a day (Goal 1). The corresponding SDG indicator is proportion of the population living below the international poverty line. To estimate the performance relative to this social foundation, we adopt this measure as our income indicator and use the World Bank data which define the poverty threshold at $1.90 for China and the World (https://data.worldbank.org/). Due to the availability of data, we choose poverty headcount ratio at national poverty line for provinces. These are obtained from *China Statistics Yearbooks* and *Poverty Monitoring Report of Rural China*. We set a threshold of 95% for this indicator, referring to O'Neill et al. (2018)[6]. We assume that values above 95% correspond to eradicating extreme poverty.

1.3.3 Water

The target in the Sustainable Development Goals is to achieve universal and equitable access to safe and affordable drinking water for all (Goal 6). The SDG indicator is proportion of population using safely managed drinking water services. The data used in our analysis are from World Bank (https://data.worldbank.org/) and *China Health Statistics Yearbooks*. A threshold value of 95% is chosen, due to the difficulties in extending universal access to the last 5% of a population, especially in very rural areas.

1.3.4 Sanitation

The target in the Sustainable Development Goals is to achieve access to adequate and equitable sanitation and hygiene for all and end open defecation (Goal 6). The SDG indicator is proportion of population using (a) safely managed sanitation services and (b) a hand-washing facility with soap and water. The data used in our study measure the percentage of the population with access to improved sanitation facilities. Our data are obtained from World Bank (https://data.worldbank.org/) and *China Health Statistics Yearbooks*. Similar to the other indicators, a threshold of 95% is selected.

1.3.5 Health care

The target in the Sustainable Development Goals is to end preventable deaths of newborns and children under 5 years of age (Goal 3). The SDG indicator is under-5 mortality rate. We use this indicator as our health care measure for China and the World obtained from World Bank (https://data.worldbank.org/). Due to the availability of data, we use under-7 health care management rate for China's provinces from *China Health Statistics Yearbooks.* Similar to the other indicators, a threshold of 95% is selected.

1.3.6 Energy

The target in the Sustainable Development Goals is to ensure universal access to affordable, reliable and modern energy services (Goal 7). The SDG indicator is proportion of population with access to electricity. Our data are from World Bank (https://data.worldbank.org/) and *China Water Statistics Yearbooks*. Similar to the other indicators, a threshold of 95% electricity access is used.

1.3.7 Education

The target in the Sustainable Development Goals is to ensure that all girls and boys complete free, equitable and quality primary and secondary education leading to relevant and effective learning outcomes (Goal 4). The SDG indicator is completion rate (primary education, lower secondary education, upper secondary education). Due to the availability of multi-scale data, adult literacy rate (ages 15 and above) is chosen as our education indicator obtained from World Bank (https://data.worldbank.org/) for China and the World. Provincial data are

obtained from *China Statistics Yearbooks (2000-2018)*. We set a threshold of 95% for education aspect, accounting for the difficulty of providing universal access to at least 5% of the population.

1.3.8 Gender equality

The SDG indicator for gender equality is number of countries with laws and regulations that guarantee full and equal access to women and men aged 15 years and older to sexual and reproductive health care, information and education (Goal 5). We adopt this measure as gender equality indicator and select the gender parity index of education, obtained from Word Bank (https://data.worldbank.org/) and *China Statistics Yearbooks (2000-2018)*. Gender parity index of education is the ratio of females to males (ages 15 and above) who can both read and write with understanding a short simple statement about their everyday life. We choose a threshold value of 1 for this indicator.

1.3.9 Social equity

The target in the Sustainable Development Goals is to empower and promote the social, economic and political inclusion of all, irrespective of age, sex, disability, race, ethnicity, origin, religion or economic or other status (Goal 10). We choose the Gini coefficients as our indicator of social equity. The data are from World Bank (https://data.worldbank.org/), *China Statistics Yearbooks* and the data from Tian et al. (2015)[20]. A threshold of 0.30 is chosen for the Gini coefficient, referring to O'Neill et al. (2018)[6]. To be consistent with the convention of higher value on social indicators representing better performance, we use the results of one minus the Gini coefficient as our measure. Thus, threshold is set as 0.70.

1.3.10 Jobs

The target in the Sustainable Development Goals is to achieve full and productive employment and decent work for all women and men, including for young people and persons with disabilities, and equal pay for work of equal value (Goal 8). We measured jobs as one minus the unemployment rate. Unemployment rate data are obtained from World Bank (https://data.worldbank.org/) and *China Statistics Yearbooks*. We set a threshold of 94% for this indicator, referring to O'Neill et al. (2018) [6]. This level is roughly equivalent to the average non-accelerating inflation rate of unemployment (NAIRU) for OECD countries.

1.4 Coupling coordination degree model

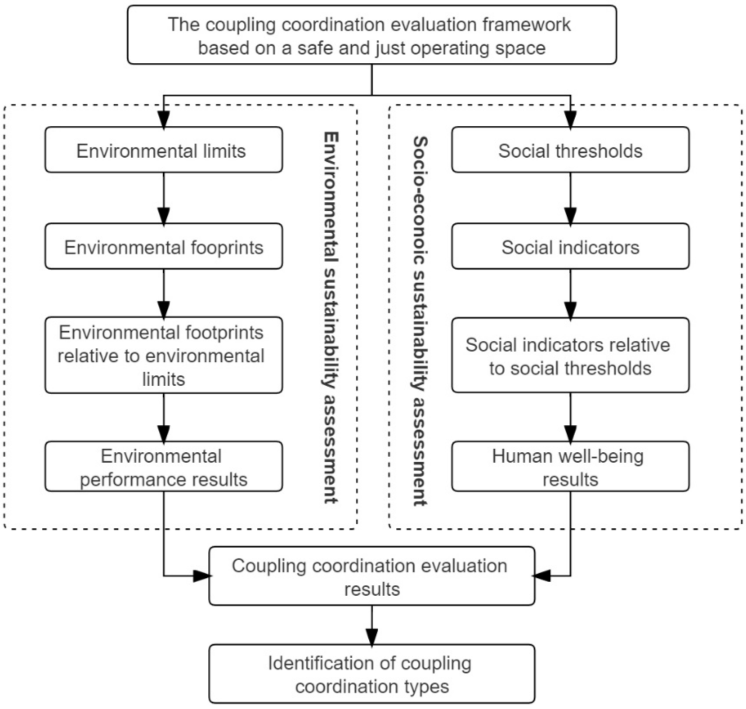

Fig. S2| The coupling coordination evaluation framework based on a safe and just operating space.

In order to capture the characteristics of the different coupling coordination degree in different regions, we adapt the standards of the coupling coordination degree given by Shi et al. (2020)[21] and Li et al. (2022)[22] and divided the magnitude of CCD into six levels. To further distinguish the performance in social and environmental aspects, we divided the CCD into three types: environmental lag, social lag, and socio-environmental synchronization. The results are shown in Table. S5:

Supplementary Table 5. Level of the coupling coordination degree.

Category	Level	Subcategory	Function	Type
Coordinated development	0.8<D≤1	High coordination	E<S	Environmental lag
			E>S	Social lag
			E=S	Socio-environmental synchronization
Transformative development	0.6<D≤0.8	Moderate coordination	E<S	Environmental lag
			E>S	Social lag
			E=S	Socio-environmental synchronization
	0.5<D≤0.6	Primary coordination	E<S	Environmental lag
			E>S	Social lag
			E=S	Socio-environmental synchronization
	0.4<D≤0.5	Basic coordination	E<S	Environmental lag
			E>S	Social lag
			E=S	Socio-environmental synchronization
Uncoordinated development	0.2<D≤0.4	Intermediate unbalanced	E<S	Environmental lag
			E>S	Social lag
			E=S	Socio-environmental synchronization
	0<D≤0.2	Extreme unbalanced	E<S	Environmental lag
			E>S	Social lag
			E=S	Socio-environmental synchronization

Note: E represents environmental performance results. S represents human wellbeing results. E=S means to |E-S|≤0.1.
Source: Shi et al., 2020[21] and Li et al., 2022[22].

1.5 Data sources

Supplementary Table 6. Data sources for the environmental indicators.

Indicator	Specific indicator	Data source
CO_2 emission	Yearly CO_2 emissions	*Carbon Emission Accounts & Datasets (CEADS)*
Blue water footprint	Freshwater use	*China Environmental Statistics Yearbooks*
Land footprint	The surface of land related to human activities	*China Land and Resources Statistical Yearbooks*
Nitrogen footprint	Allocation of nitrogen fertilizer applied to cropland	*China Rural Statistical Yearbooks*
Phosphorus footprint	Allocation of phosphorus fertilizer applied to cropland	*China Rural Statistical Yearbooks*

Supplementary Table 7. Data sources for the social indicators.

Indicator	Specific indicator	Data source
Food security	Percentage of undernourished Children under 5	*China Health Statistics Yearbooks*
Income	Poverty headcount ratio at national poverty lines	*China Statistics Yearbooks* and *Poverty Monitoring Report of Rural China*
Water	Households with piped water	*China Health Statistics Yearbooks*
Sanitation	Households with access to improved sanitation facilities	*China Health Statistics Yearbooks*
Health care	Percentage of health care management for children under 7	*China Health Statistics Yearbooks*
Education	Percentage of illiterate population to total aged 15 and over	*China Statistics Yearbooks*
Energy	Percentage of electricity access	*China Water Statistics Yearbooks*
Gender equality	Education gap between women and men	*China Statistics Yearbooks*
Social equity	Gini coefficient	*China Statistics Yearbooks* and Tian et al. (2015)[20]
Jobs	Unemployment rate	*China Statistics Yearbooks*

Supplementary Table 8. Data sources for the driving factors.

	Driving factors	Data source
Economic factors	Real gross Regional Product (GDP) (in 2000 prices)	*China Statistics Yearbooks*
	Fixed capital investment	*China Statistics Yearbooks*
	Primary industry product	*China Statistics Yearbooks*
	Secondary industry product	*China Statistics Yearbooks*
	Tertiary industry product	*China Statistics Yearbooks*
Social factors	Population	*China Statistics Yearbooks*
	Real household consumption (in 2000 prices)	*China Statistics Yearbooks*
	Urban population density	*China Statistics Yearbooks*
	Urbanization rate	*China Statistics Yearbooks*
	Illiteracy rate	*China Statistics Yearbooks*
Environmental factors	NDVI	Resource and Environment Science and Data Center (https://www.resdc.cn/)
	Annual precipitation	Resource and Environment Science and Data Center
	Average annual temperature	Resource and Environment Science and Data Center
	Sunshine hours	Resource and Environment Science and Data Center
	Relative humidity	Resource and Environment Science and Data Center
	Crop area	Resource and Environment Science and Data Center
	Forest area	Resource and Environment Science and Data Center
	Grass area	Resource and Environment Science and Data Center
	Build area	Resource and Environment Science and Data Center
	Total energy consumption	*China Energy Statistics Yearbooks*
	Agricultural fertilizer	*China Statistics Yearbooks*
	Total water use	*China Environmental Statistics Yearbooks*
	Environmental invest (cumulative investments)	Bryan et al., 2018[23]
	Sustainability programme (cumulative areas)	Bryan et al., 2018[23]

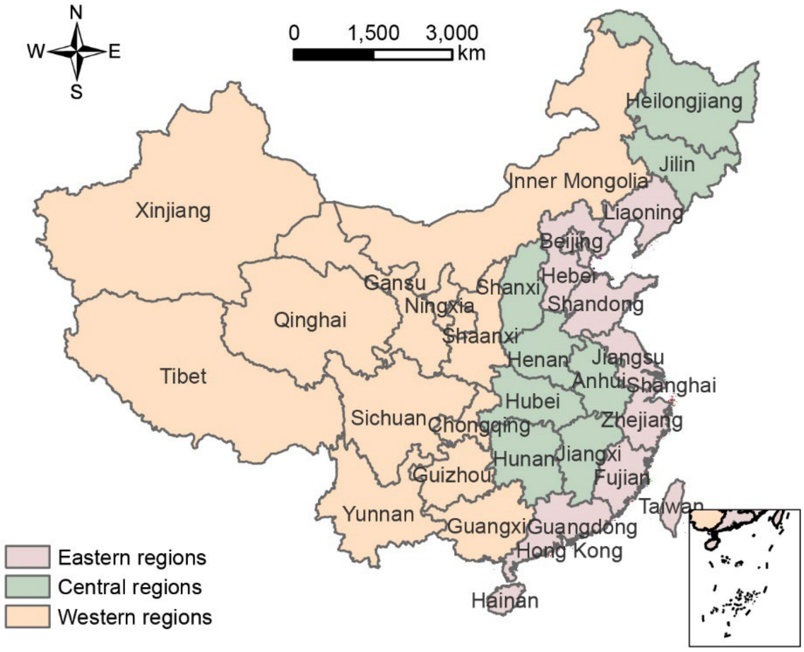

Fig. S3|Spatial distribution of the study areas.

1.7 Key definitions

Supplementary Table 9. Definitions of key concepts used in this article.

	**Definition**
Social-ecological system	Social–ecological systems are integrated systems of humans and nature that constitute a complex adaptive system with ecological and social components that interact dynamically through various feedbacks[24,25].
Planetary boundary	The planetary boundary framework proposes quantitative global limits to nine anthropogenic perturbation of crucial Earth System processes. Within the nine boundaries, humanity can continue to develop and thrive for generations to come. If these boundaries are crossed, then important subsystems could shift into a new state, often with deleterious or potentially even disastrous consequences for humans[1].
Environmental footprint	Environmental footprints reflect human pressure on the environment in relation to resource extractions and waste emissions.
Environmental performance	Environmental performance represents the development level of the ecological system, calculated as the ratio of environmental footprints to corresponding environmental limits (downscaled planetary boundaries).
Human well-being	Human well-being represents the development level of the human system, calculated as the ratio of social indicators to corresponding social thresholds.
Coupling coordination degree	"Coupling" refers to the phenomenon that two or more systems interact with each other closely in various ways. "Coordination" reflects the degree of coherence between all subsystems, as well as the extent to which the system tends to be ordered. Coupling coordination degree is a measure of the synergies among subsystems, which determines the development trend of integrated system from disorder to order.
Coupling	High values of coupling coordination degree depict synergies between environmental performance and human well-being.
Decoupling	Low values of coupling coordination degree depict trade-offs between environmental performance and human well-being.
Environmental development lag	Environmental performance below human well-being.
Social development lag	Environmental performance above human well-being.
Environmental-social synchronization	Environmental performance matches human well-being.
Synergy	Synergies are suggested if both indicators increase over time (quadrat I), i.e., positive correlations over time.
Trade-off	Tradeoffs are suggested if one indicator increases as the other decreases (quadrat II, III), i.e., negative correlations over time.
Lose-lose	If both indicators decline, lose-lose outcomes are suggested (quadrat IV).
The level of coupling	The level of coupling is quantified by the coupling coordination degree.

The level of development	The level of development is quantified by the changing trends in coupling coordination degree.
Coupled	The level of coupling is above the dividing point.
Uncoupled	The level of coupling is below the dividing point.
Developed	The level of development is above the dividing point. The regions or systems classified as developed tend to have an increasing level of coupling.
Underdeveloped	The level of development is below the dividing point. The regions or systems classified as developed tend to have a decreasing level of coupling.

2 Supplementary results

2.1 Per-capita environmental limits

Supplementary Table 10. Per capita environmental limits of China from 2000 to 2018, compared to the respective environmental footprints.

Years	Climate change t/capita	Freshwater use m^3/capita	Land-system change ha/capita	Nitrogen cycle kg/capita	Phosphorus cycle kg/capita
2000	1.73	654	0.33	6.49	1.014
2001	1.72	646	0.32	6.41	1.001
2002	1.70	638	0.32	6.32	0.988
2003	1.69	630	0.31	6.25	0.976
2004	1.67	622	0.31	6.17	0.964
2005	1.65	614	0.31	6.09	0.952
2006	1.63	607	0.30	6.02	0.940
2007	1.60	599	0.30	5.94	0.929
2008	1.58	592	0.30	5.87	0.917
2009	1.55	585	0.29	5.80	0.906
2010	1.53	578	0.29	5.73	0.896
2011	1.50	571	0.28	5.67	0.885
2012	1.47	564	0.28	5.60	0.875
2013	1.44	558	0.28	5.53	0.865
2014	1.41	551	0.27	5.47	0.855
2015	1.38	545	0.27	5.41	0.845
2016	1.35	539	0.27	5.34	0.835
2017	1.32	533	0.27	5.28	0.825
2018	1.29	527	0.26	5.22	0.816

2.2 Comparison of consumptive and territorial environmental performance

2.2.1 Climate change

Fig. S4 shows that China does not operate within the boundary for climate change during 2000-2018, both measured in territorial and consumptive terms. A somewhat surprising result is that the difference between territorial and consumptive emissions is smaller than the amount beyond the boundary. In other words, China tends to perform by the same order of magnitude relative to the downscaled PB, whether measured in territorial or consumptive terms.

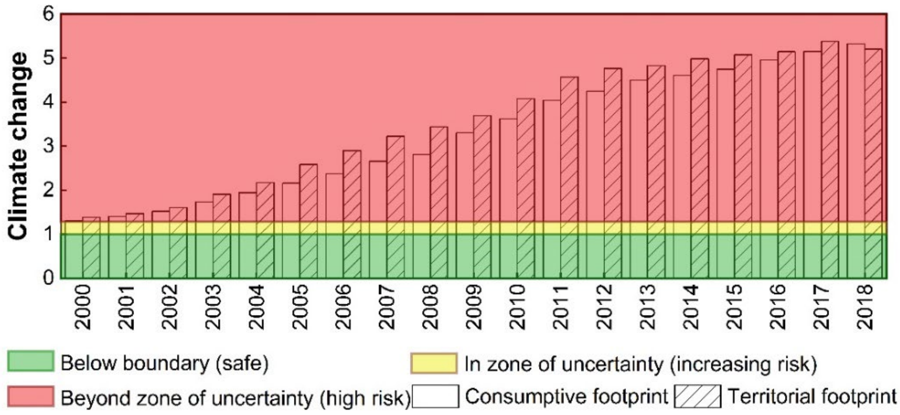

Fig. S4| Comparison of consumptive and territorial performance relative to per capita climate change boundary (i.e., ratio of carbon footprint to fare-share climate change boundary). Background colors show the zones of downscaled environmental limit. Vertical bars represent consumption-based and production-based CO_2 emissions against fair-share environmental limits over time.

2.2.2 Freshwater use

Fig. S5 shows that China operates within the per capita boundary for freshwater use from 2000 to 2018. A territorial measure is higher than a consumptive measure. This can be explained by the fact that China tends to produce water-intensive goods and services.

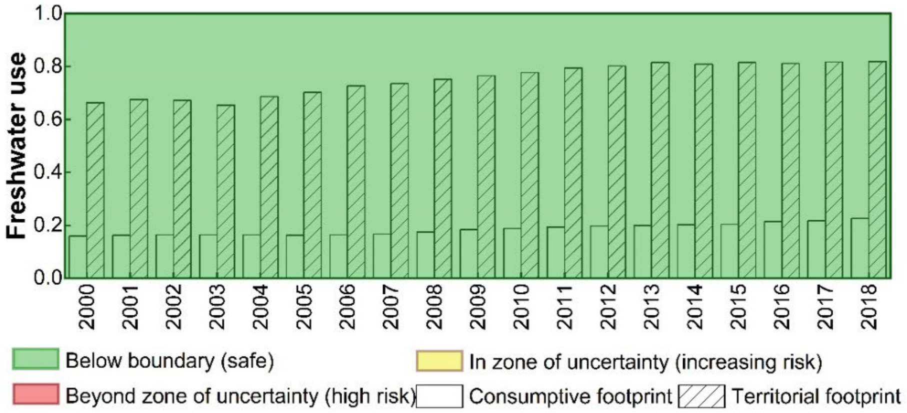

Fig. S5| Comparison of consumptive and territorial performance relative to per capita freshwater use boundary (i.e., ratio of blue water footprint to fare-share freshwater use boundary). Background colors show the zones of downscaled environmental limit. Vertical bars represent consumption-based and production-based blue water use against fair-share environmental limits over time.

2.2.3 Land-system change

Fig. S6 shows consumptive and territorial performance on the per capita land boundary. China does not transgress the consumption-based boundary for land-system change during 2000-2018. The global land migration embodied in trade links the cropland footprints of countries of agri-food production to countries of consumption. Although China is responsible for agricultural and food exports in the world trade, it performs better on consumptive measures. The reason can be that as the most populous country, China's consumption is not yet land-intensive, with low levels of national land scarcity.

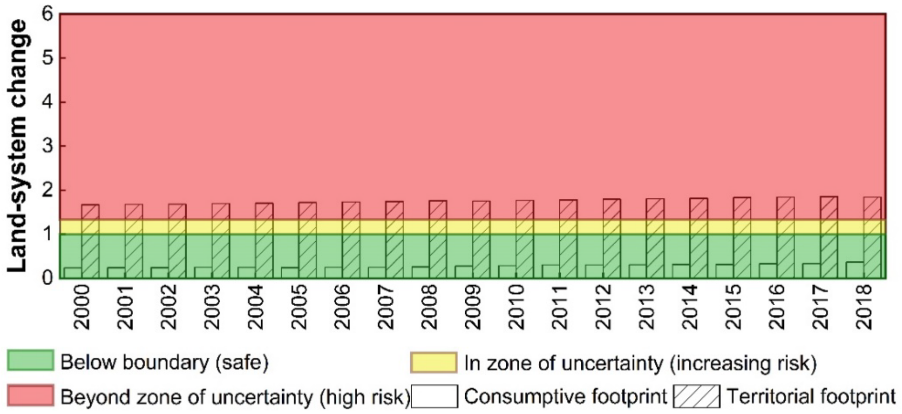

Fig. S6| Comparison of consumptive and territorial performance relative to per capita land-system change boundary (i.e., ratio of land footprint to fare-share land-system change boundary). Background colors show the zones of downscaled environmental limit. Vertical bars

represent consumption-based and production-based land footprints against fair-share environmental limits over time.

2.2.4 Biogeochemical flows

Fig. S7 and S8 show that China does not operate within the boundary for nitrogen and phosphorus cycle during 2000-2018, both measured in territorial and consumptive terms. In addition, territorial footprints are much higher than consumptive footprints in China. The difference between territorial and consumptive performance is larger for nitrogen and phosphorus than for climate change (Fig. S4, S7, and S8). China has relatively limited per capita consumptive use of nitrogen and phosphorus, whereas territorial footprint is likely to show much higher per capita use, probably due to the large proportion of China's agricultural and food exports.

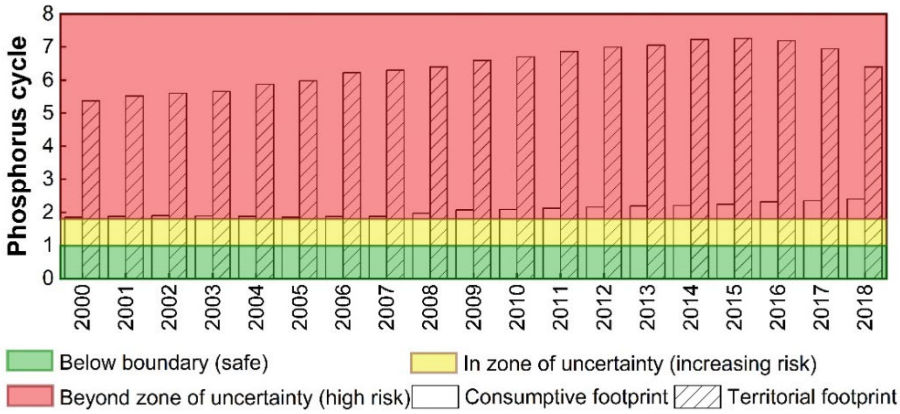

Fig. S7| Comparison of consumptive and territorial performance relative to per capita phosphorus cycle boundary (i.e., ratio of phosphorus footprint to fare-share phosphorus cycle boundary). Background colors show the zones of downscaled environmental limit. Vertical bars represent consumption-based and production-based phosphorus footprints against fair-share environmental limits over time.

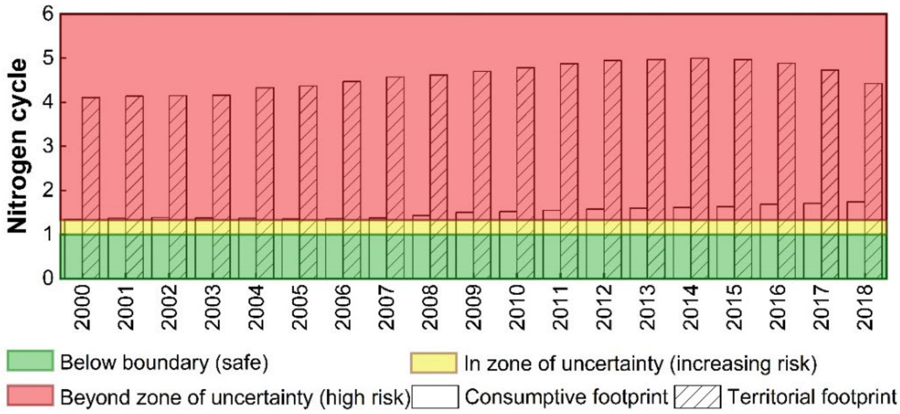

Fig. S8| Comparison of consumptive and territorial performance relative to per capita nitrogen cycle boundary (i.e., ratio of nitrogen footprint to fare-share nitrogen cycle boundary). Background colors show the zones of downscaled environmental limit. Vertical bars represent consumption-based and production-based nitrogen footprints against fair-share environmental limits over time.

2.3 Additional indicators for measuring environmental performance

Policy-oriented assessments need to address sustainability issues from regional to global in various socio-ecological contexts. By considering environmental performance at multiple scales, we are more likely to link regional and global sustainability realistically and effectively[26]. From a

regional context perspective, we have added additional indicators related to environmental quality, referring to key aspects of national policy concerns. There are good data available on state changes with respect to the environment, such as air quality, water quality, and resource use.

2.3.1 Air quality

Indicators characterizing the air quality are: $PM_{2.5}$ concentration, ratio of good air quality days in prefecture-level and above cities, etc. Considering that WHO specifies guideline values for air pollutants such as $PM_{2.5}$, so we chose the annual average concentration of $PM_{2.5}$ as a measure of the air quality. To ensure the consistency of the time series, we derived the annual average $PM_{2.5}$ concentration (V5.GL.01) in China's 30 provinces from 2000 to 2018 from the Atmospheric Composition Analysis Group[27]. This dataset was generated by combining a chemical transport model, remote sensing data and monitoring data, which considers both the coverage and accuracy of the estimates for $PM_{2.5}$ concentration[28].

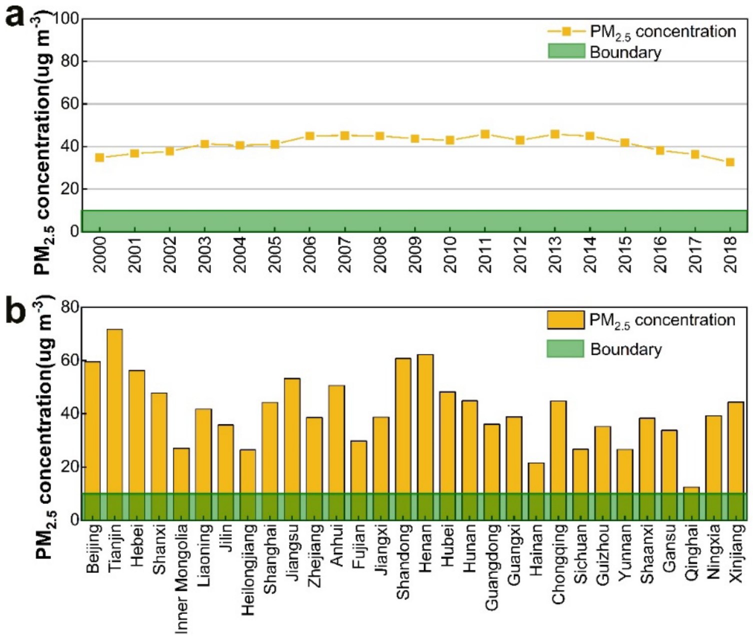

Fig. S9| Performance on the air quality in China. a. Temporal trends of $PM_{2.5}$ concentration in China from 2000 to 2018. b. Spatial changes in $PM_{2.5}$ concentration in 30 provinces (average of 2000-2018).

China released air quality standard for ambient $PM_{2.5}$ (35 ug/m^3 for annual mean concentration) (GB3095-2012)[29], referring to the World Health Organization (WHO) Air Quality Guidelines of 10 µg/m^3 [30]. The latest Global Air Quality Standards Guidelines further revised the concentration limits of annual average $PM_{2.5}$ concentration to 5 µg/m^3 [31]. However, more than 99% of the population is exposed to concentrations in excess of the World Health Organization (WHO) Air Quality Guidelines of 10 µg/m^3 [32,33]. Thus, a threshold value of 10 µg/m^3 is chosen, due to the difficulties in reaching the WHO 2005 AQG guideline[30].

The Chinese government implemented the Air Pollution Prevention and Control Action Plan in 2013, which has a significant impact on reducing $PM_{2.5}$ concentration[34]. As shown in Fig. S9.a, substantial improvements in air quality have been observed since 2013, with $PM_{2.5}$ concentration in 2018 reduced by 28% compared to 2013. However, all provinces have not reached the WHO guideline (Fig. S9.b). 73% of the provinces are still exposed to annual mean concentrations of $PM_{2.5}$ that exceed 35 µg/m^3 (Fig. S9.b). Hence, to achieve the target of SDG

13, stronger air quality control policy is still required to make substantial further reductions in air pollution[35]).

2.3.2 Water quality

Indicators characterizing the quality of the water environment are: the proportion of surface water reaching or better than Class III water bodies, the proportion of poor V water bodies in surface water, etc. Considering that the proportion of poor V water bodies in surface water has been reduced to 6.7% in 2018, so we set the proportion of surface water to reach or better than III water body as a measure of the water quality, from *Ministry of Ecology and Environment of China* (https://www.mee.gov.cn/hjzl/sthjzk/zghjzkgb/). As the target of "13th Five-Year Plan", a threshold of 70% is chosen. Due to the availability of data, we choose the waste water treatment rate as the measure of water quality at the provincial scale, from *China Environmental Statistics Yearbook*. A threshold value of 95% is chosen, due to the difficulties in extending universal access to the last 5% of a population.

As shown in Fig. S10.a, substantial improvements in water quality have been observed from 2001 to 2018. At the provincial level, water quality still needs to be improved in all provinces (Fig. S10.b).

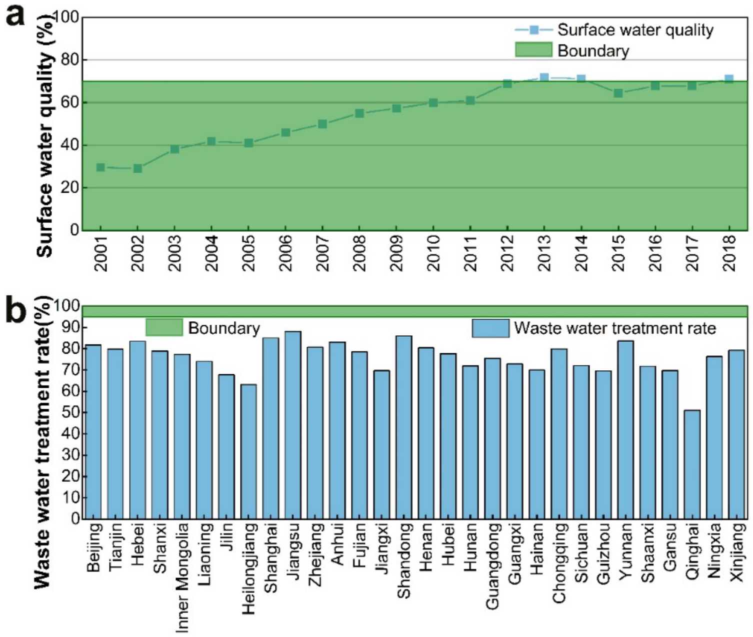

Fig. S10| Performance on the water quality in China. a. Temporal trends of surface water quality in China from 2001 to 2018. b. Spatial changes in waste water treatment in 30 provinces (average from 2000 to 2018).

2.3.3 Resource use

We choose the indicators of energy use per unit of GDP and water use per unit of GDP (in constant 2000 price) as the measure of resource use, from *China Energy Statistics Yearbook* and *China Environmental Statistics Yearbook*. Energy use per unit of GDP is the amount of energy consumed by a country (region) for each unit of GDP produced in a certain period, reflecting energy use efficiency. Water consumption per unit of GDP refers to the amount of water used per unit of GDP produced, reflecting water resources utilization.

As shown in Fig. S11.a and S12.a, substantial improvements in resource use efficiency have been found from 2000 to 2018. At provincial scale, resource consumption varies greatly among provinces (Fig. S11.b and S12.b).

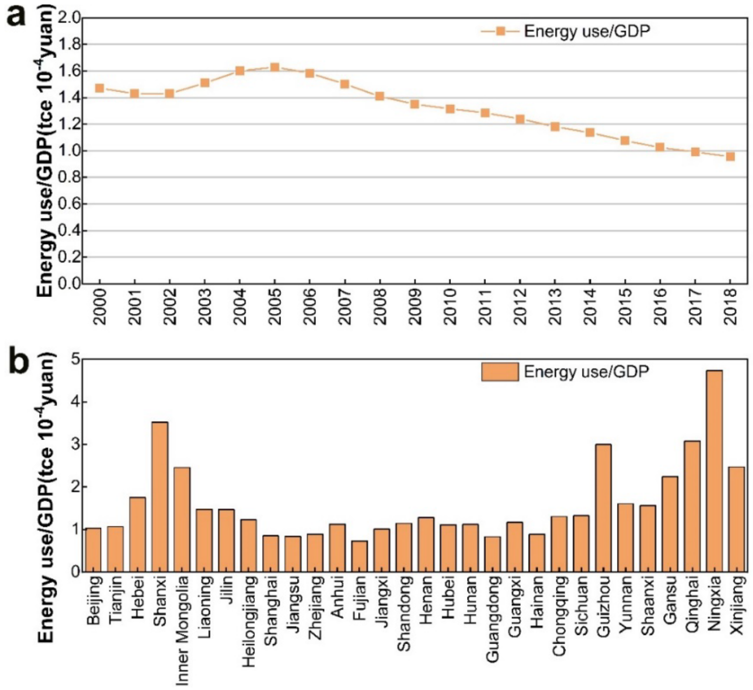

Fig. S11| Performance on energy consumption in China. a. Temporal trends of energy consumption per GDP in China from 2000 to 2018. b. Spatial changes in energy consumption per GDP in 30 provinces (average from 2000 to 2018).

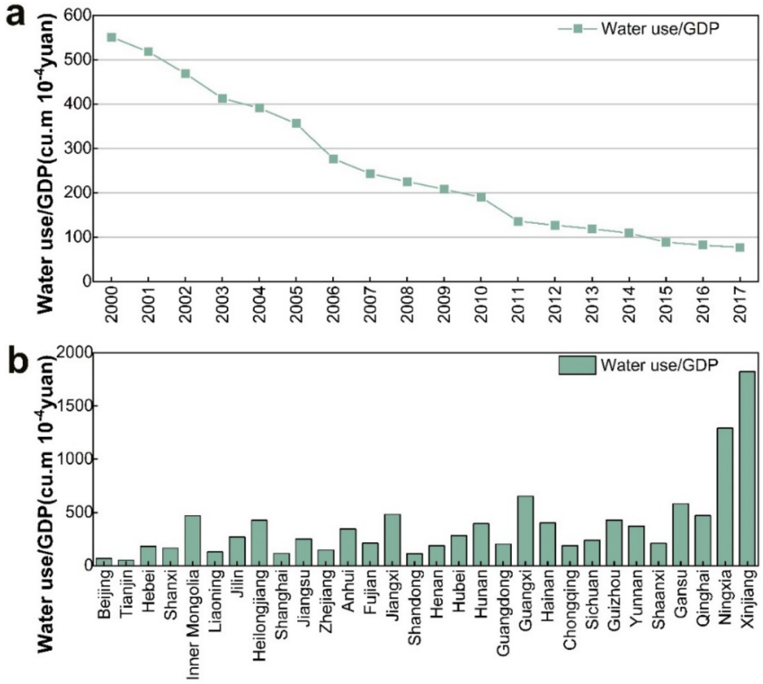

Fig. S12| Performance on water use in China. a. Temporal trends of water use per GDP in China from 2000 to 2017. b. Spatial changes in water use per GDP in 30 provinces (average from 2000 to 2018).

2.4 Performance relative to environmental limits and social foundations

Supplementary Table 11. Performance with respect to per capita environmental limits.

Environmental indicator	Global footprint	China footprint	Fair-share boundary	Unit
Climate change	4.70	5.95	1.54	Ton CO_2 per year
Freshwater use	381	425	587	Cubic meter H_20 per year
Land-system change	0.22	0.10	0.29	Hectare per year
Nitrogen cycle	14.24	21.44	5.82	Kilogram N per year
Phosphorus cycle	5.88	9.03	0.91	Kilogram P per year

Supplementary Table 12. Performance with respect to social thresholds.

Social indicator	Global	China	Threshold	Unit
Food security	87.34	87.41	95	% nourished rate (0-5 years old)
Income	84.10	88.36	95	% who earn above poverty headcount
Water	70.09	92.39	95	% with access to piped water
Sanitation	43.86	68.28	95	% with access to improved sanitation facilities
Health care	45.04	80.76	95	% health care management rate, children under 7
Energy	83.06	98.98	95	% with access to electricity
Education	83.68	92.71	95	% literacy rate, adult total (aged 15 and above)
Gender equality	0.929	0.899	1	literacy rate, adult total (aged 15 and above), gender parity index (GPI)
Social equity	-	52.71	70	(1 - Gini Index) * 100
Jobs	94.25	95.99	94	% of labor force employed

2.5 Trends of environmental performance and human well-being

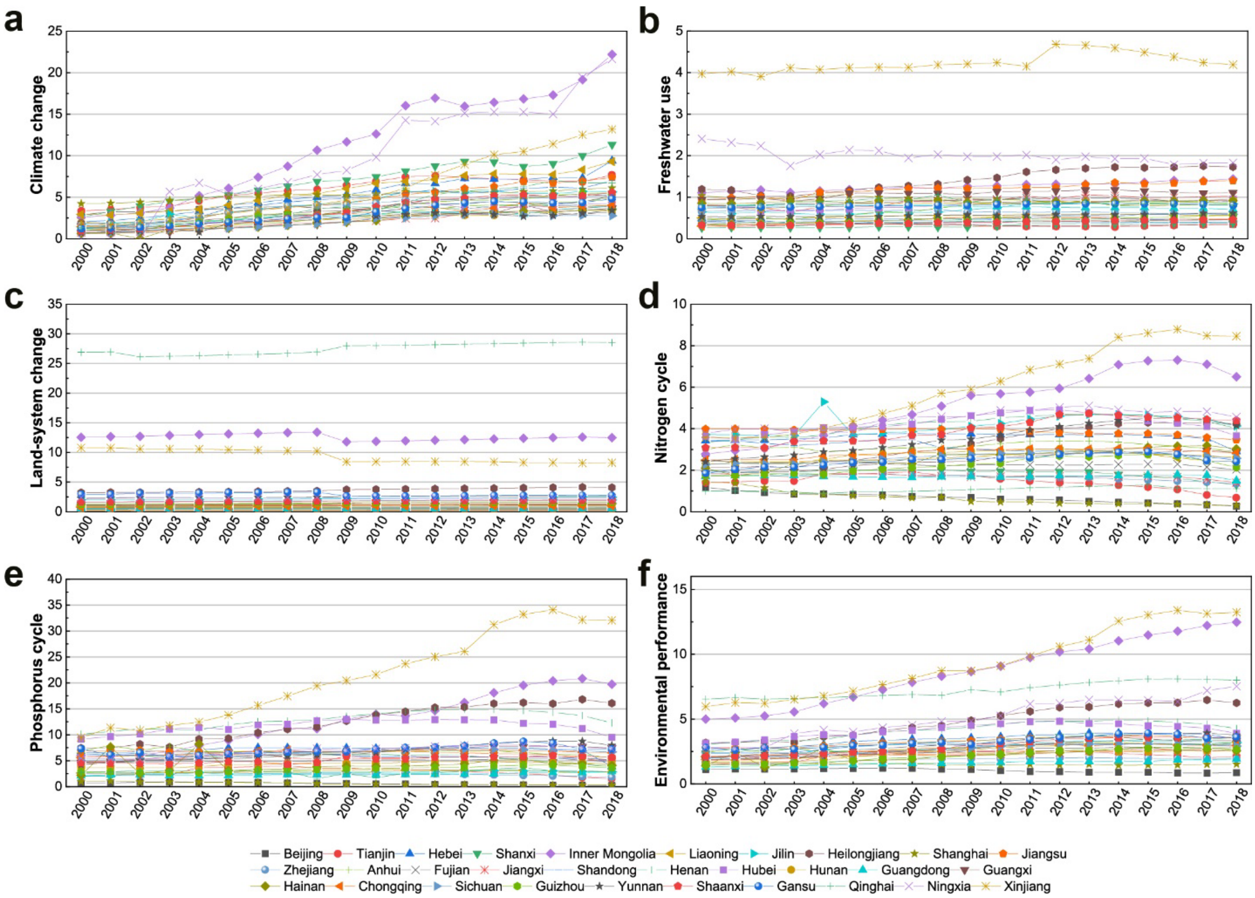

Fig. S13|Temporal trends of environmental performance in China's 30 provinces from 2000 to 2018. The y-axes represent the ratio of environmental footprints to corresponding planetary boundaries. The unit is scale.

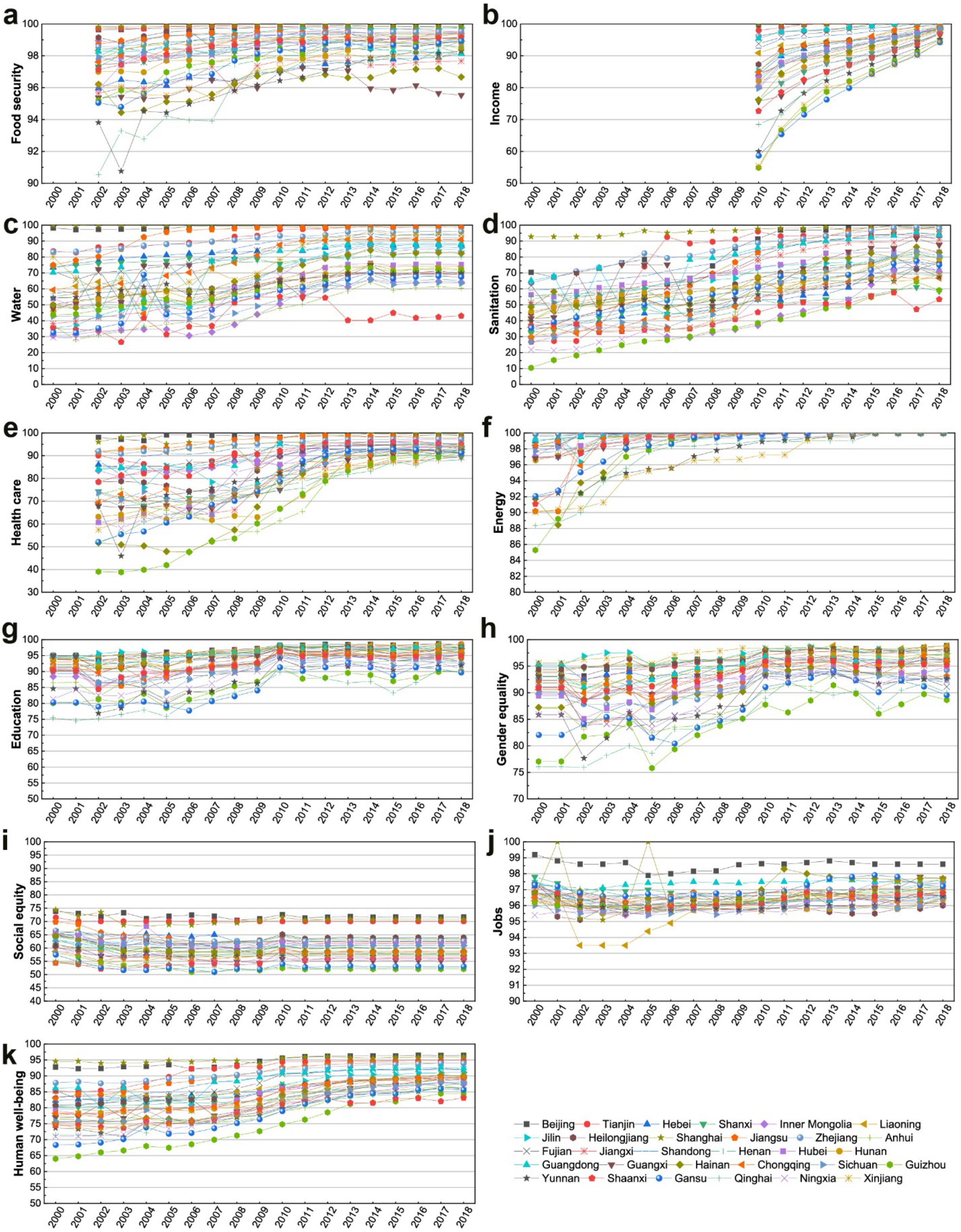

Fig. S14|Temporal trends of human well-being in China's 30 provinces from 2000 to 2018. The y-axes represent the ratio of social indicators to corresponding social thresholds. The unit is scale.

Supplementary Table 13. Changing slopes of performance of environmental footprints in China.

Regions	Climate change	Freshwater use	Land-system change	Nitrogen cycle	Phosphorus cycle
**China**	**0.257***	**0.010***	**0.011***	0.040*	**0.125***
Beijing	-0.017	-0.005*	-0.004*	-0.042*	-0.042*
Tianjin	0.265*	-0.001	-0.005*	-0.055*	-0.076
Hebei	0.384*	-0.002*	0.005*	0.003	0.008
Shanxi	0.454*	0.007*	0.008*	-0.019*	-0.035
Inner Mongolia	1.091*	0.017*	-0.011	0.289*	0.928*
Liaoning	0.366*	0.006*	0.013*	0.026*	0.037*
Jilin	0.253*	0.019*	0.026*	0.060*	0.055*
Heilongjiang	0.236*	0.048*	0.056*	0.154*	0.609*
Shanghai	0.095*	-0.014*	-0.002*	-0.048*	-0.044*
Jiangsu	0.352*	0.024*	0.003*	-0.019*	-0.044*
Zhejiang	0.204*	-0.005*	0.002*	-0.028*	-0.036*
Anhui	0.232*	0.027*	0.010*	0.012	-0.046*
Fujian	0.250*	0.011*	0.007*	-0.012*	0.038*
Jiangxi	0.182*	0.017*	0.009*	-0.008*	0.032
Shandong	0.312*	0.000	0.004*	-0.045*	-0.046*
Henan	0.192*	0.008*	0.009*	0.077*	0.299*
Hubei	0.172*	0.017*	0.015*	0.062*	0.135*
Hunan	0.160*	0.009*	0.011*	0.028*	0.067*
Guangdong	0.139*	-0.006*	-0.001*	0.002	0.029*
Guangxi	0.188*	0.011*	0.028*	0.071*	0.195*
Hainan	0.183*	0.004*	0.008*	0.067*	0.118*
Chongqing	0.176*	0.010*	0.008*	0.030*	0.060*
Sichuan	0.142*	0.013*	0.023*	0.033*	0.145*
Guizhou	0.259*	0.011*	0.026*	0.066*	0.088*
Yunnan	0.153*	0.004*	0.024*	0.136*	0.273*
Shaanxi	0.285*	0.008*	0.018*	0.101*	0.095*
Gansu	0.213*	0.006*	-0.011	0.057*	0.153*
Qinghai	0.336*	-0.003*	0.119*	0.017*	0.009
Ningxia	1.079*	-0.025*	-0.009	0.074*	0.156*
Xinjiang	0.661*	0.025*	-0.1658	0.349*	1.465*

Note: the given significance level α is 0.05 (*).

Supplementary Table 14. Changing slopes of human well-being in China.

Regions	Food security	Income	Water	Sanitation	Health care	Energy	Education	Gender equality	Social equity	Jobs
**China**	**0.023***	**0.040***	**0.026***	**0.027***	**0.023***	**0.009***	**0.020***	**0.016***	**0.005**	**0.000**
Beijing	0.003*	0.001*	0.002*	0.026*	0.001	0.000	0.012*	0.013*	-0.012	0.000
Tianjin	-0.003*	0.001*	0.018*	0.040*	0.009*	0.000	0.015*	0.015*	0.000	0.000*
Hebei	0.031*	0.025*	0.013*	0.024*	0.012*	0.000*	0.016*	0.009*	-0.016*	0.001
Shanxi	0.022*	0.049*	0.006*	0.017*	0.028*	0.003*	0.012*	0.009*	-0.016*	-0.001*
Inner Mongolia	0.005*	0.036*	0.037*	0.027*	0.016*	0.001*	0.025*	0.019*	-0.004	0.002*
Liaoning	0.013*	0.020*	0.017*	0.025*	0.003	0.000*	0.013*	0.011*	-0.014	0.003*
Jilin	0.012*	0.025*	0.057*	0.022*	0.013*	0.000*	0.008*	0.005	0.000	0.002*
Heilongjiang	-0.004	0.022*	0.020*	0.024*	0.024*	0.000*	0.012*	0.009*	0.010	0.000
Shanghai	0.000	0.000	0.000*	0.005*	0.004*	0.000	0.012*	0.015*	-0.002	0.001
Jiangsu	0.006*	0.009*	0.020*	0.052*	0.008*	0.000*	0.019*	0.022*	-0.036*	0.002*
Zhejiang	0.015*	0.009*	0.015*	0.023*	0.008*	0.000*	0.023*	0.018*	-0.020*	0.003*
Anhui	0.012*	0.033*	0.034*	0.018*	0.026*	0.001*	0.028*	0.027*	-0.020	0.003*
Fujian	0.026*	0.014*	0.030*	0.040*	0.013*	0.001*	0.025*	0.023*	-0.021*	0.001*
Jiangxi	0.027*	0.036*	0.035*	0.032*	0.025*	0.008*	0.015*	0.017*	-0.025*	0.000
Shandong	0.008	0.015*	0.043*	0.027*	0.003	0.000	0.019*	0.017*	0.000	0.000
Henan	0.043*	0.027*	0.017*	0.018*	0.028*	0.007*	0.013*	0.012*	-0.012	0.000
Hubei	0.015*	0.031*	0.032*	0.022*	0.039*	0.003*	0.020*	0.020*	-0.015*	0.003*
Hunan	0.027*	0.043*	0.026*	0.020*	0.038*	0.003*	0.015*	0.015*	-0.012*	0.001
Guangdong	-0.001	0.009*	0.025*	0.022*	0.015*	0.002*	0.010*	0.013*	-0.011	0.001*
Guangxi	0.008	0.055*	0.013	0.033*	0.034*	0.013*	0.014*	0.017*	-0.023*	0.003*
Hainan	0.034*	0.040*	0.039*	0.027*	0.061*	0.016*	0.018*	0.021*	0.000	0.002
Chongqing	0.018*	0.026*	0.041*	0.027*	0.030*	0.005*	0.020*	0.017*	0.001	0.001*
Sichuan	0.015*	0.033*	0.038*	0.041*	0.033*	0.008*	0.015*	0.011*	-0.006	0.001*

Guizhou	0.047*	0.085*	0.030*	0.033*	0.071*	0.025*	0.031*	0.028*	-0.033*	0.002*
Yunnan	0.072*	0.065*	0.018*	0.020*	0.034*	0.040*	0.034*	0.030*	-0.001*	0.001
Shaanxi	0.021*	0.056*	0.019*	0.015*	0.021*	0.007*	0.022*	0.017*	0.013	0.001
Gansu	0.055*	0.089*	0.041*	0.028*	0.054*	0.019*	0.039*	0.029*	-0.007	0.002*
Qinghai	0.090*	0.070*	0.026*	0.009*	0.041*	0.028*	0.044*	0.041*	-0.012	0.002*
Ningxia	0.030*	0.045*	0.067*	0.039*	0.047*	0.000*	0.034*	0.026*	-0.021*	0.001*
Xinjiang	0.042*	0.081*	0.037*	0.033*	0.038*	0.046*	0.013*	0.006*	0.019*	0.003*

Note: the given significance level α is 0.05 (*).

2.6 Relationships between environmental performance and human well-being

We further analyzed spatial and temporal variations in complex interactions between environmental performance and human well-being based on the method in Qiu et al., 2018[36]. In brief, static relationships are analyzed using the average values for each province during 2000-2018; for temporal dynamics in relationships, we calculated the changes in relationships using the changing slopes of coupling coordination degree from 2000 to 2018.

2.6.1 Spatial variations in relationships

Relationships between performance on environmental and socio-economic aspects are represented by numbers of social foundations reached and numbers of environmental limits operated within for China's provinces (Fig. S15.a), and comprehensive development levels of human well-being and environmental performance (Fig. S15.b).

In general, at the provincial scale, the areas where more environmental footprints are within planetary boundaries tend to achieve more social thresholds as well (Fig. S15.a). Notably, several provinces perform well both on the environmental and social aspects, such as Beijing, Shanghai, and Tianjin. In this way, there is generally a positive correlation between human well-being and environmental performance at the provincial scale. Specifically, the more social thresholds a province achieves, the more environmental limits it operates within, and vice versa. For example, Shanghai has achieved 9 social foundations and only transgressed 1 environmental boundary. Whereas, Ningxia, Xinjiang, Guangxi, and Inner Mongolia have transgressed all 5 environmental limits, with only 3 social indicators above the thresholds. For normalized results (Fig. S15.b), the relationship is not statistically significant. There is insignificant positive correlation between human well-being and environmental performance, except for Xinjiang, Qinghai, and Inner Mongolia. Environmental performance indicates the ratio of environmental footprints to downscaled planetary boundaries (i.e., average across all five environmental indicators). Human well-being indicates the ratio of social indicators to social thresholds (i.e., average across all ten social indicators).

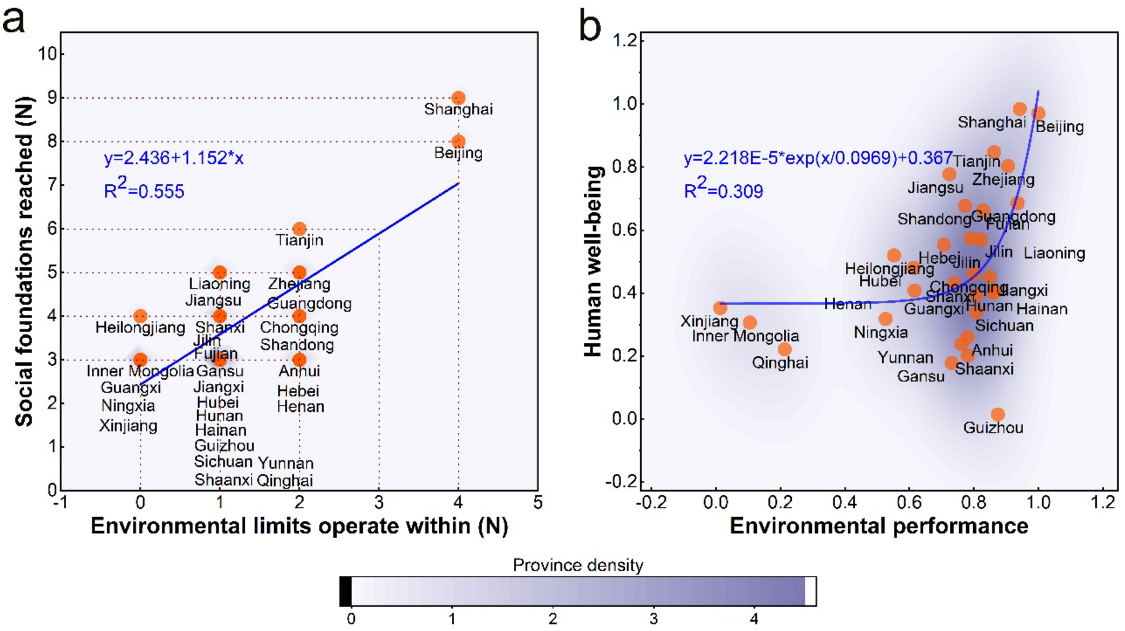

Fig. S15| Relationships between performance on environmental and socio-economic aspects. (a) represents relationships between numbers of social foundations reached and numbers of environmental limits operated within for China's provinces, and (b) reflects relationships between comprehensive development levels of human well-being and environmental performance. The shade represents the kernel density of provinces. The best-fit curve and comparable R^2 value are shown on each plot.

2.6.2 Temporal variations in relationships

Dynamic changes in interactions indicate that relationships between human well-being and environmental performance change over time and vary among provinces (Fig. S16). In specific, trade-offs over time between human well-being and climate change, freshwater use, land-system change, nitrogen cycle, and phosphorus cycle appear in 47%, 50%, 56%, 60%, and 50% of the provinces (Fig. S16.a-e). For relationships between human well-being (i.e., average across all ten social indicators) and comprehensive environmental performance (i.e., average across all five environmental indicators) (Fig. S16.f), 47% of the provinces exhibit synergies (i.e., positive correlations over time), mainly located in eastern China (Fig. S17.f). Whereas 43% of the provinces show trade-offs (i.e., negative correlations over time), mainly located in western and central China (Fig. S17.f).

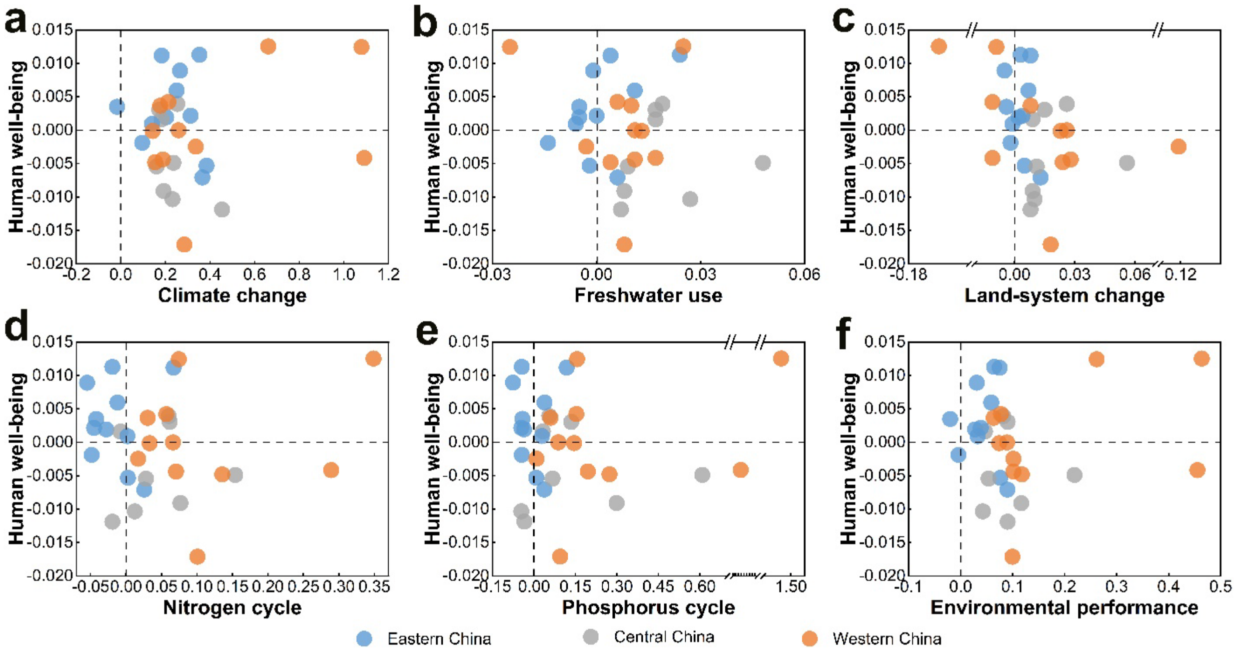

Fig. S16| Changes in relationships between performance on socio-economic and environmental aspects from 2000 to 2018. Colored circles represent 30 provinces. Temporal changes in indicators are calculated using Sen' slope at the provincial scale. Human well-being indicates the ratio of social indicators to social thresholds (i.e., average across all ten social indicators). Quadrant I represent synergies, Quadrants II and IV trade-offs, and Quadrant III lose-lose outcomes. Synergies are suggested if both indicators increase over time, and trade-offs are suggested if one indicator increases as the other decreases. If both decrease, lose-lose outcomes are suggested.

Supplementary Table 15. Changes in relationships between human well-being and environmental performance for China's provinces from 2000 to 2018.

	Climate change	Freshwater use	Land-system change	Nitrogen cycle	Phosphorus cycle	Environmental performance
Synergies	47%	30%	30%	26%	33%	47%
Tradeoffs	47%	50%	56%	60%	50%	43%
Lose-lose	0%	10%	7%	7%	10%	3%
No trend	6%	10%	7%	7%	7%	7%

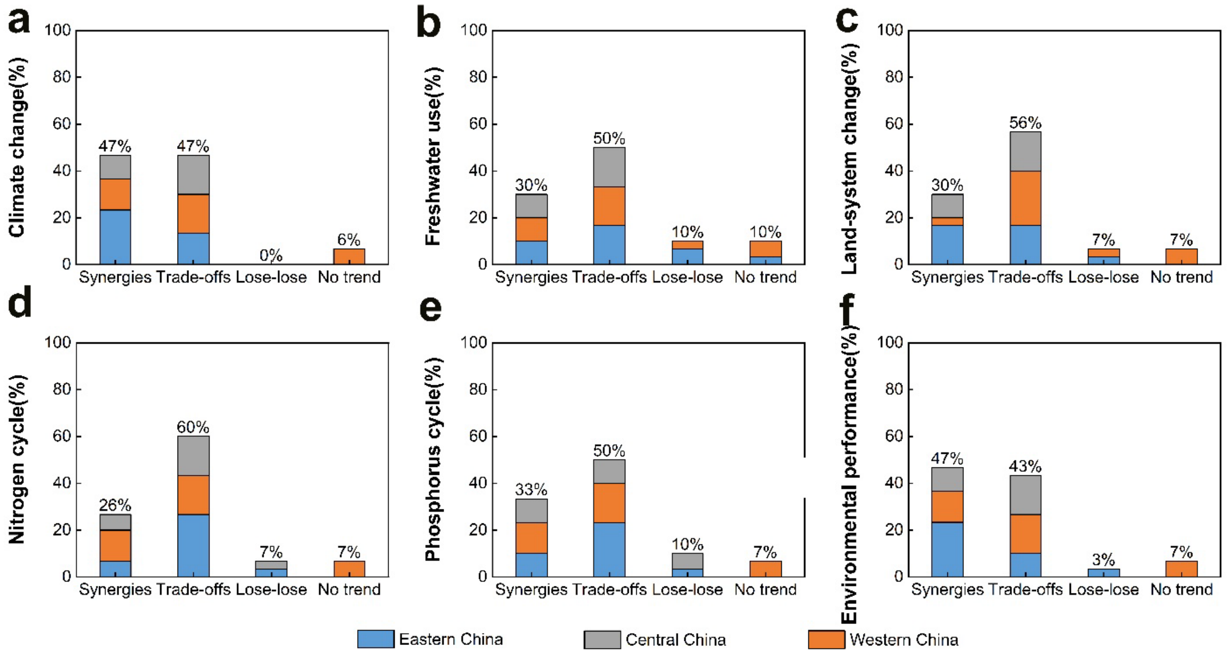

Fig. S17| Changes in relationships between human well-being and environmental performance for China's provinces from 2000 to 2018.

2.7 Coupling coordination relationships

2.7.1 Magnitudes and changing trends of coupling coordination degree

Supplementary Table 16. Coupling coordination degree between environmental performance and human well-being for China's provinces from 2000 to 2018.

Regions	Environmental performance	Human well-being	CCD value	Lag type	Z value of CCD	Slope of CCD
Beijing	1.000	0.971	0.993	Environmental-social Synchronized	3.231*	0.00100
Tianjin	0.863	0.847	0.923	Environmental-social Synchronized	4.408*	0.00316
Hebei	0.707	0.554	0.790	Social lag	-2.239*	-0.00173
Liaoning	0.822	0.570	0.827	Social lag	-4.268*	-0.00373
Shanghai	0.943	0.985	0.982	Environmental-social Synchronized	-1.749	-0.00032
Jiangsu	0.724	0.777	0.865	Environmental-social Synchronized	5.458*	0.00513
Zhejiang	0.906	0.804	0.924	Social lag	2.029*	0.00036
Fujian	0.829	0.662	0.860	Social lag	2.729*	0.00249
Shandong	0.772	0.678	0.850	Environmental-social Synchronized	2.029*	0.00199
Guangdong	0.935	0.686	0.893	Social lag	-0.770	-0.00041
Hainan	0.862	0.406	0.765	Social lag	3.429*	0.00570
**Eastern region**	**0.851**	**0.722**	**0.879**	**Social lag**	**3.499***	**0.00156**
Inner Mongolia	0.105	0.307	0.394	Environmental lag	-1.679	-0.00923
Guangxi	0.806	0.392	0.746	Social lag	-1.679	-0.00366
Chongqing	0.799	0.462	0.778	Social lag	2.799*	0.00239
Sichuan	0.807	0.339	0.723	Social lag	0.140	0.00024
Guizhou	0.874	0.015	0.094	Social lag	3.017*	0.00000
Yunnan	0.761	0.238	0.650	Social lag	-2.379*	-0.00439
Shaanxi	0.781	0.202	0.516	Social lag	-4.080*	-0.02038
Gansu	0.731	0.178	0.597	Social lag	3.289*	0.00491
Qinghai	0.213	0.222	0.373	Environmental-social Synchronized	4.572*	0.02296
Ningxia	0.526	0.318	0.632	Social lag	3.359*	0.00518
Xinjiang	0.013	0.353	0.075	Environmental lag	-3.215*	0.00000
**Western region**	**0.583**	**0.275**	**0.507**	**Social lag**	**-0.980**	**-0.00103**
Shanxi	0.740	0.431	0.749	Social lag	-4.688*	-0.00539
Jilin	0.790	0.574	0.820	Social lag	3.429*	0.00129
Heilongjiang	0.552	0.520	0.731	Environmental-social Synchronized	-4.478*	-0.00359
Anhui	0.781	0.260	0.663	Social lag	-2.589*	-0.00592
Jiangxi	0.850	0.451	0.786	Social lag	0.770	0.00054
Henan	0.616	0.408	0.706	Social lag	-3.778*	-0.00324
Hubei	0.614	0.480	0.736	Social lag	3.848*	0.00465
Hunan	0.862	0.402	0.766	Social lag	-2.099*	-0.00375
**Central region**	**0.726**	**0.441**	**0.745**	**Social lag**	**-2.799***	**-0.00266**
**China**	**0.720**	**0.483**	**0.707**	**Social lag**	**0.070**	**0.00003**

Note: the given significance level α is 0.05 (*).

2.7.2 Drivers of changes in coupling coordination degree

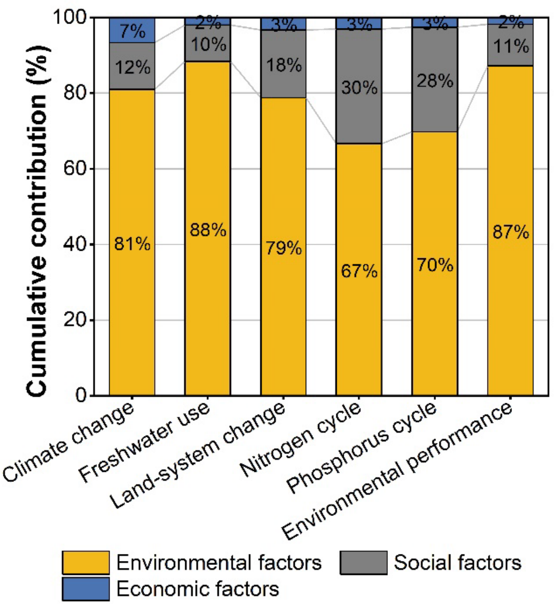

Fig. S18| Cumulative contributions of driving factors to changes in coupling coordination degrees between environmental performance and aggregated human well-being. For each indicator, we show the contribution of each of the three factors towards the changes in coupling coordination degrees: environmental (yellow), social (grey), and economic (blue) factors.

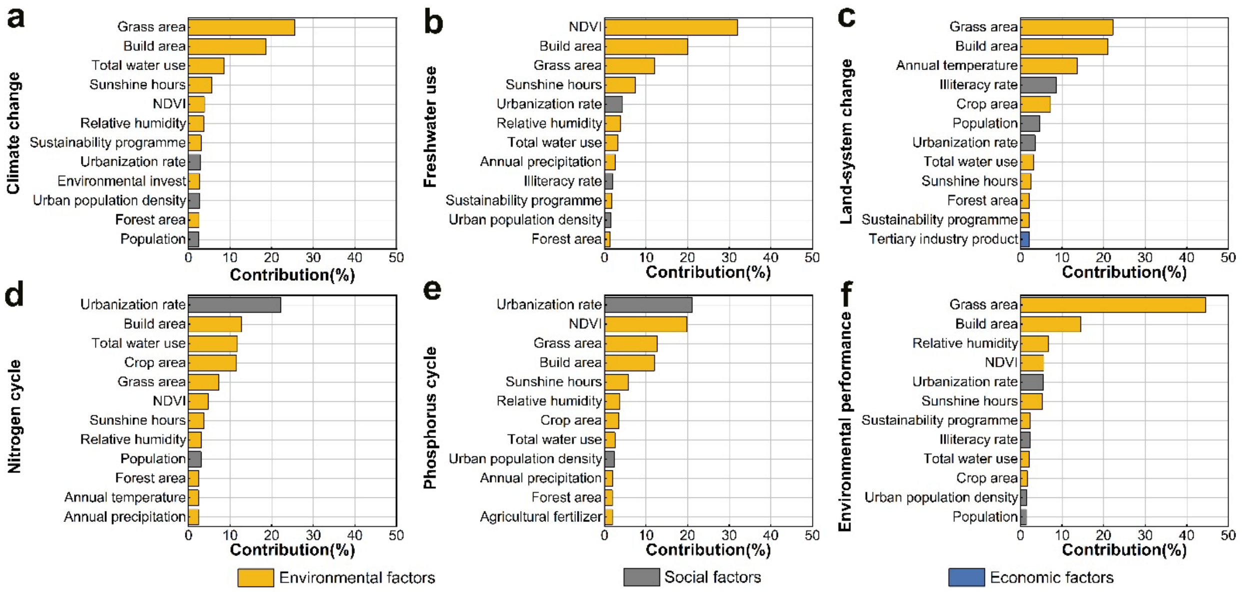

Fig. S19| Contribution of driving factors to changes in coupling coordination degrees between environmental performance and human well-being. Shown are the relative contributions, as integer percentage (%). Coupling coordination degree between aggregated human well-being and (a) climate change, (b) freshwater use, (c) land-system change, (d) nitrogen cycle, (e) phosphorus cycle, and (f) overall environmental performance. For each indicator, we show the contribution of each of the three factors towards the changes in coupling coordination degrees: environmental (yellow), social (grey), and economic (blue) factors.

Supplementary Table 17. Cumulative contributions of driving factors to changes in coupling coordination degrees between environmental performance and human well-being in China.

	Driving factors	Climate change	Fresh water use	Land-system change	Nitrogen cycle	Phosphorus cycle	Environmental performance
Economic factors	Fixed capital investment	2.05	0.64	0.46	0.39	0.67	0.34
	Primary industry product	1.34	0.32	0.10	0.89	0.51	0.59
	Real GDP	0.76	0.40	0.47	0.41	0.32	0.36
	Secondary industry product	0.45	0.23	0.18	0.22	0.19	0.16
	Tertiary industry product	2.07	0.42	2.08	1.15	0.92	0.31
Social factors	Illiteracy rate	1.86	2.04	8.58	2.25	1.22	2.27
	Population	2.51	1.16	4.61	3.00	1.85	1.40
	Real household consumption	2.41	0.43	0.27	0.60	1.17	0.25
	Urban population density	2.74	1.59	0.94	2.33	2.30	1.51
	Urbanization rate	2.82	4.38	3.54	22.15	21.03	5.55
Environmental factors	Agricultural fertilizer	0.83	0.39	1.70	1.70	1.85	1.02
	Annual precipitation	1.90	2.64	0.38	2.35	1.93	0.52
	Annual temperature	0.52	1.18	13.65	2.38	1.18	0.98
	Build area	18.57	19.98	21.02	12.79	12.00	14.50
	Crop area	2.29	1.21	7.25	11.57	3.51	1.62
	Environmental invest	2.75	0.82	0.57	0.77	0.97	0.68
	Forest area	2.57	1.40	2.13	2.49	1.86	1.00
	Grass area	25.56	12.11	22.19	7.32	12.65	44.58
	NDVI	3.84	31.96	0.76	4.67	19.81	5.57
	Relative humidity	3.65	3.79	0.96	3.03	3.74	6.81
	Sunshine hours	5.57	7.49	2.52	3.73	5.84	5.25
	Sustainability programme	3.05	1.66	2.12	1.15	1.14	2.33
	Total energy consumption	1.31	0.46	0.36	0.91	0.85	0.35
	Total water use	8.59	3.29	3.16	11.79	2.50	2.06

Fig. S20 shows the changes in the influence of the dominant drivers, indicating the change in their influence on coupling coordination degrees with the change in the value of the driver.

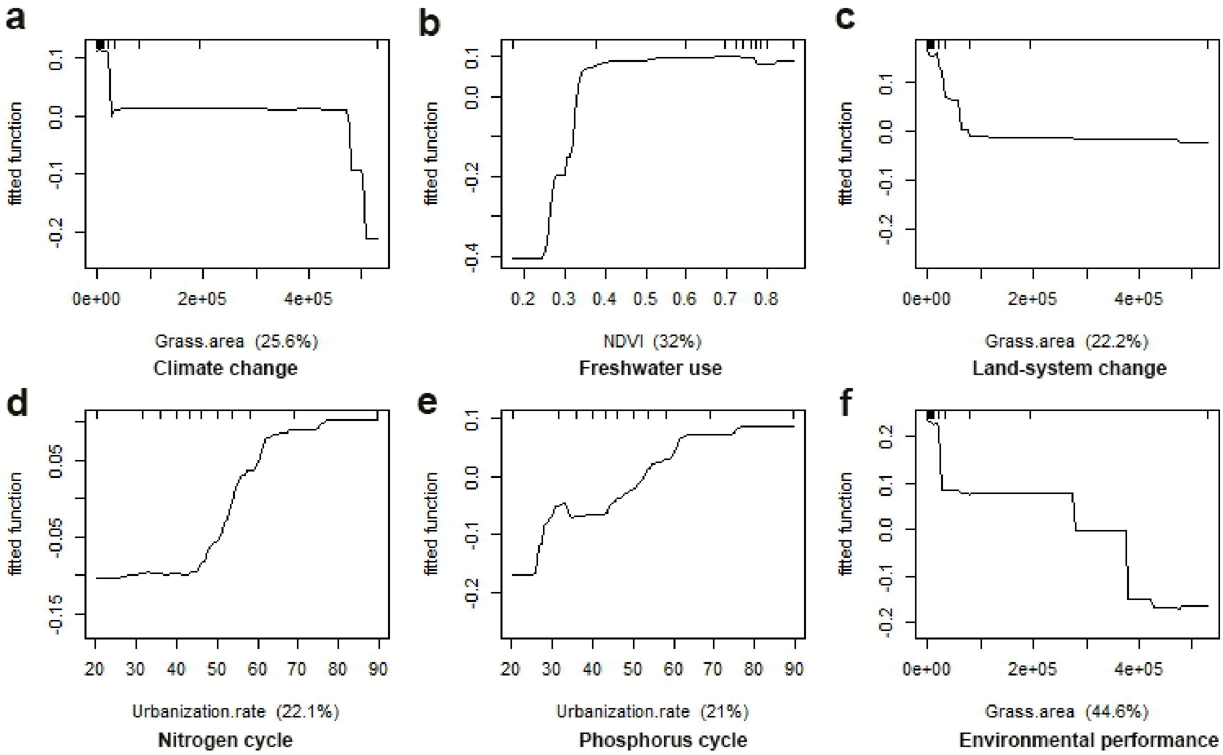

Fig. S20| The influence of drivers on coupling coordination degrees between environmental performance and aggregated human well-being. Coupling coordination degree between

aggregated human well-being and (a) climate change, (b) freshwater use, (c) land-system change, (d) nitrogen cycle, (e) phosphorus cycle, and (f) environmental performance. Where a relative impact value greater than zero indicates that the driver is positively correlated with CCD, less than zero indicates a negative correlation, and a value of zero indicates that there is no correlation between the two. The short line in the upper border of each graph is the decile scale, indicating the range of each 10% data point.

2.8 Characteristics and drivers of development patterns

Supplementary Table 18. Characteristics of four development patterns.

Development pattern	Level of coupling(C)	Level of development (D)	Lag type	Province
Quadrant I	C>0.8	D>0	Environmental-social Synchronized	Beijing, Tianjin, Jiangsu, Shandong
			Social lag	Jilin, Zhejiang, Fujian
Quadrant II	C>0.8	D<0	Social lag	Liaoning, Guangdong
			Environmental-social Synchronized	Shanghai
Quadrant III	C<0.8	D<0	Social lag	Hebei, Shanxi, Anhui, Henan Hunan, Guangxi, Yunnan, Shaanxi
			Environmental-social Synchronized	Heilongjiang
			Environmental lag	Inner Mongolia, Xinjiang
Quadrant IV	C<0.8	D>0	Social lag	Jiangxi, Hubei, Hainan, Chongqing Sichuan, Guizhou, Gansu, Ningxia
			Environmental-social Synchronized	Qinghai

Supplementary Table 19. Driving factors associated with coupling coordination degree in the four development patterns. Mean values and changing slopes of drivers are reported outside and inside parentheses during the period 2000-2018, respectively.

Category	Independent variable	Quadrant I	Quadrant II	Quadrant III	Quadrant IV
Environmental	Annual precipitation	973.72(+4.23*)	1213.61(+4.23*)	823.11(+4.23*)	1035.4(+4.23*)
variables	Average annual temperature	13.61(+5.09*)	15.56(+5.00*)	11.71(+5.18*)	12.91(+5.18*)
	Sunshine hours	213.42(+2.3*)	205.44(+2.39*)	217.86(+4.28*)	198.62(+5*)
	Relative humidity	66.5(-0.77)	70.8(-0.32)	63.99(-0.95)	66.77(-0.77)
	Build area	8239.04(-4.23*)	7085.94(-4.23*)	7917.54(+4.23*)	2274.36(-4.23*)
	Crop area	43760.94(-4.23*)	37688.23(-1.85)	85357.12(+0.43)	46941.92(+3.28*)
	Forest area	35230.88(-3.75*)	55203.38(-3.28*)	100243.77(-4.23*)	64250.63(-4.23*)
	Grass area	6438(-0.68)	5694.88(-1.67)	120574.8(+0.14)	85714.35(-0.14)
	NDVI	0.74(+4.64*)	0.7(+5.36*)	0.67(+5.36*)	0.63(+5.09*)
	Total water use	189.2(+4.37*)	235.91(+1.49)	236.07(+4.28*)	129.4(+4.01*)
	Total energy consumption	12479.42(+5.36*)	15831.08(+5.36*)	11202.46(+5.36*)	6288.97(+5.36*)
	Agricultural fertilizer	173.47(+0.86)	121.71(+0.14)	232.08(+0.32)	111.68(-0.23)
	Sustainability programme	6735.07(+5.36*)	7659.85(+5.36*)	34427.37(+5.36*)	44366.88(+5.36*)
	Fixed capital invest	22660.88(+5.36*)	18634.54(+5.27*)	31930.55(+5.36*)	24395.11(+5.36*)
Social	Population	4509.15(+5.36*)	5380.94(+5.36*)	4903.64(+4.55*)	3259.22(+3.83*)
variables	Urbanization rate	60.54(+5.36*)	69.77(+4.82*)	41.53(+5.36*)	40.91(+5.36*)
	Urban population density	1712.77(+3.74*)	2255.05(+4.64*)	2524.81(+3.56*)	2209.53(+3.56*)
	Environmental invest	6.51(-1.22)	4.18(-1.13)	7.3(-2.39*)	11.28(-1.4)
	Illiteracy rate	9815.93(-3.29*)	9135.2(-3.74*)	6982.97(-3.47*)	4352.16(-3.29*)
Economic	Real GDP (in 2000 prices)	12996.33(+5.36*)	16340.34(+5.36*)	7115.55(+5.36*)	4400.43(+5.36*)
variables	Primary industry product	816.43(+5.36*)	748.58(+5.36*)	803.98(+5.36*)	502.37(+5.36*)
	Secondary industry product	7213.34(+5.36*)	9157.01(+5.36*)	3961.56(+5.36*)	2498.45(+5.36*)
	Tertiary industry product	4962(+5.36*)	6362.09(+5.36*)	2390.21(+5.36*)	1478.7(+5.36*)

	Real household consumption (in 2000 prices)	10603.4(+5.27*)	15671.54(+5.36*)	5627.84(+5.36*)	5259.26(+5.36*)

Note: the given significance level α is 0.05 (*).

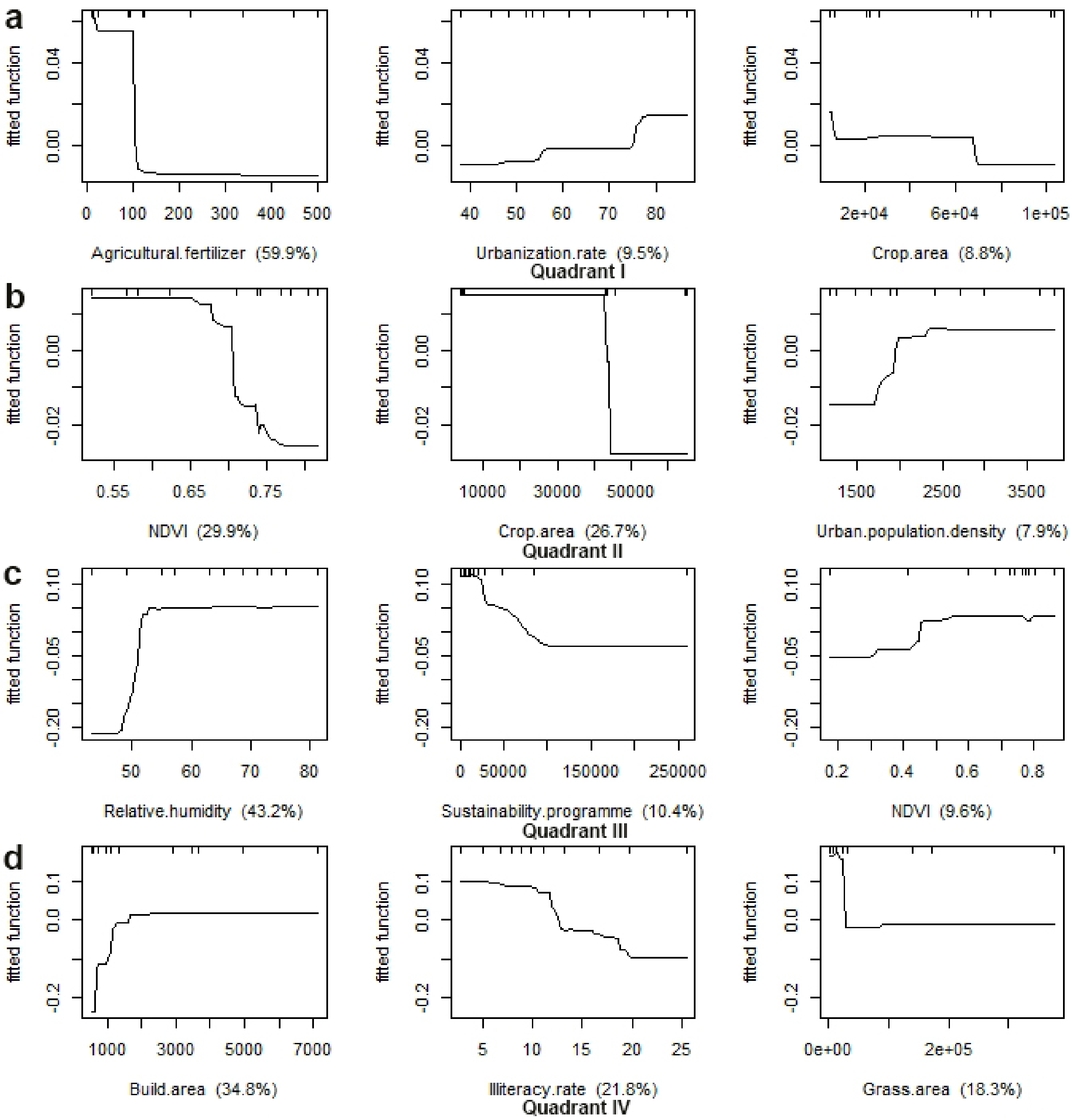

Fig. S21| The influence of drivers on coupling coordination degrees between environmental performance and human well-being in four development patterns. Where a relative impact value greater than zero indicates that the driver is positively correlated with CCD, less than zero indicates a negative correlation, and a value of zero indicates that there is no correlation between the two. The short line in the upper border of each graph is the decile scale, indicating the range of each 10% data point.

2.9 Sensitivity analysis

Parameter sensitivity analysis refers to the identification of sensitive factors that have a significant impact on the final results from a large number of uncertain variables, and the analysis and measurement of their degree of influence on the results and the degree of

sensitivity. Sensitivity analysis methods include local sensitivity analysis and global sensitivity analysis. Local sensitivity analysis considers only the impact of a single parameter on the results. For the global analysis, we need to consider the impact of multiple changing parameters on the results. Considering the computational capabilities integrated, we adopt the local sensitivity analysis method. The local sensitivity analysis index is represented by the following equation:

$$S_i = \frac{\Delta Y}{\Delta X}\frac{X}{Y}$$

where S_i represents the impact of parameter *X* on the simulation result of *Y*, *ΔX* denotes the variation of parameter *X* and *ΔY* denotes the variation of *Y* with parameter *X*. If *|Si|* is equal to zero, parameter changes have no impact on the results. Otherwise, parameter changes have an impact on the results. The higher the value is, the higher the impact of parameter changes on the results is.

To quantify the impact of our downscaling methods on our results, we choose the five downscaled boundaries as parameters, including climate change, freshwater use, land-system change, nitrogen cycle, and phosphorus cycle. The input variables in the present research are the above five environmental impacts, while the output variables are coupling coordination degrees. We obtained the corresponding output results when input variables varied from 0.8 times x to 1.6 times x, based on previous studies using per capita shares[6] (Nykvist et al., 2013; Hoff et al., 2014; Dao et al., 2015; O'Neill et al., 2018). The variation of parameters cannot exceed their reasonable range. Parameter sensitivity is calculated in national scale from 2000 to 2018. The results are summarized in Fig. S22.

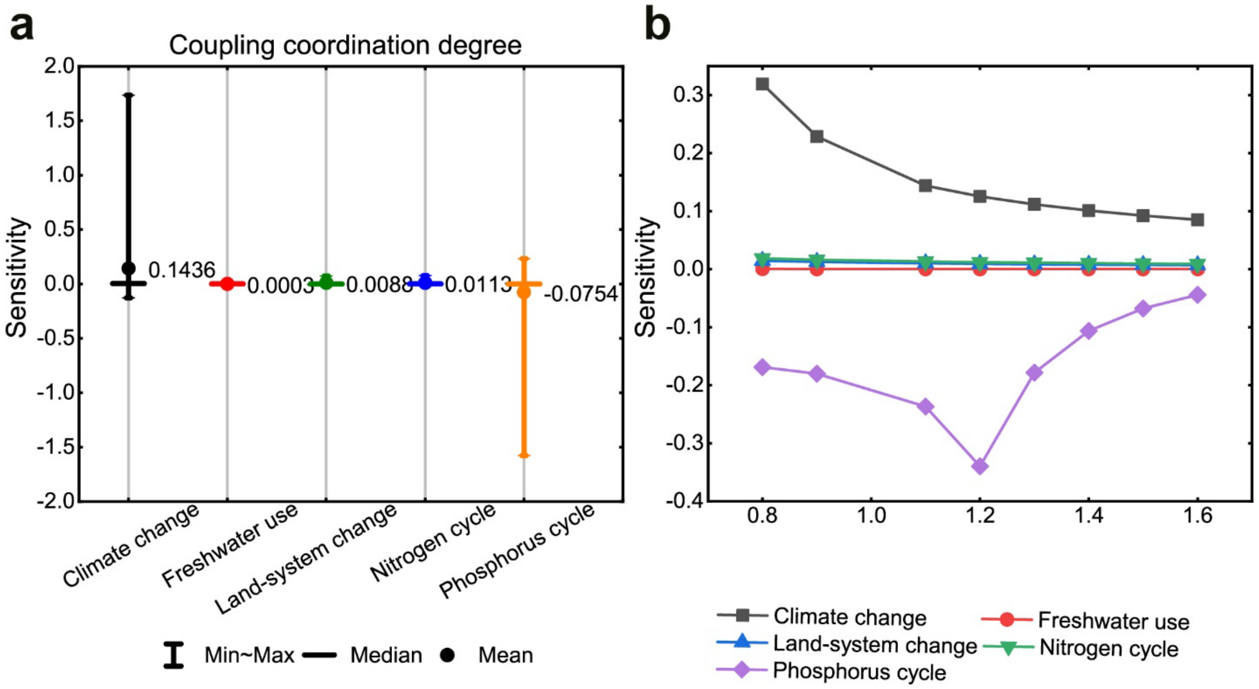

Fig. S22| Sensitivity of downscaled method to coupling coordination degree variations. (a) range of sensitivity indices (b) variations of sensitivity indices for the five per capita boundaries in China. In interval chart (from down to up), the start of the vertical line represents the minimum value, horizontal lines represent median values, the end of the vertical line represents the maximum value (*n*=years (from 2000 to 2018) =19), and the dots represent mean values.

The sensitivity indices of five per capita boundaries that have impacts on coupling coordination degree are shown in Fig. S22. We calculate the average of the parameter sensitivities in China from 2000 to 2018. The sensitivities of the four per capita boundaries are higher than 0, while the sensitivity of phosphorus cycle boundary is lower than 0 (Fig. S22.a). The most sensitivity boundary is climate change, followed by phosphorus cycle. That means downscaled climate change boundary is the most influential input variable on the coupling coordination degrees between environmental performance and human well-being.

To explore how parameter sensitivity varies with parameters, the values of parameters are taken from interval between 0.8 times and 1.6 times the default value (x). For coupling coordination degree, the sensitivity indices for freshwater use, land-system, and nitrogen cycle are relatively stable and the variation is less than 0.01 (Fig. S22.b). The sensitivity indices decrease with climate change boundary. That is, the coupling coordination is more sensitive to changes in the climate change boundary when the boundary value is relatively low (stricter limits). This means that as the population grows, the per capita boundary decreases, which may lead to a significant decrease in the coupling coordination degrees between environmental performance and human well-being. Referring to the sensitivity analysis, the contribution of downscaled climate change boundary to the variability of the coupling coordination degree is absolutely dominated at national scale. Therefore, it is recommended that policy-makers should pay more attention to the climate change dimension.

References

1. Rockström, J. *et al.* A safe operating space for humanity. *Nature* **461**, 472-475 (2009a).
2. Steffen, W. *et al.* Planetary boundaries: guiding human development on a changing planet. *Science* **347**, 1259855 (2015).
3. Turner II, B. L. *et al.* Two types of global environmental change: definitional and spatial-scale issues in their human dimensions. *Glob. Environ. Chang* **1**, 14-22 (1990).
4. Fang, K., Wang, S., He, J., Song, J. & Jia, X. Mapping the environmental footprints of nations partnering the belt and road initiative. *Resour. Conserv. Recycl.* **164**, 105068 (2021).
5. Chen, X., Li, C., Li, M. & Fang, K. Revisiting the application and methodological extensions of the planetary boundaries for sustainability assessment. *Sci. Total Environ.* **788**, 147886 (2021).
6. O'Neill, D. W., Fanning, A. L., Lamb, W. F. & Steinberger, J. K. A good life for all within planetary boundaries. *Nat. Sustain.* **1**, 88-95 (2018).
7. Fang, K., Heijungs, R. & Snoo, G. D. Understanding the complementary linkages between environmental footprints and planetary boundaries in a footprint–boundary environmental sustainability assessme nt framework. *Ecol. Econ.* **114**, 218-226 (2015).
8. Shaikh, M. A., Hadjikakou, M. & Bryan, B. A. National-level consumption-based and production-based utilisation of the land-system change planetary boundary: patterns and trends. *Ecol. Indic.* **121**, 106981 (2020).
9. Häyhä, T., Lucas, P. L., Vuuren, D. V., Cornell, S. E. & Hoff, H. From Planetary Boundaries to national fair shares of the global safe operating space — How can the scales be bridged? *Glob. Environ. Chang* **40**, 60-72 (2016).
10. Nykvist, B. *et al.* National Environmental Performance on Planetary Boundaries. (Swedish Environmental Protection Agency, 2013).

11. IPCC. *Climate Change 2014: Synthesis Report*. Contribution of Working Groups I, II, and III to the Fifth Assessment Report of the Intergovernmental Panel on Climate Change (IPCC, Geneva, Swizerland, 2014).

12. EDGAR. *Emissions Database for Global Atmospheric Research*. Fossil CO_2 and GHG emissions of all world countries - 2019 Report, EUR 29849 EN. ISBN 978-92-76-11100-9, doi:10.2760/687800, JRC117610 (Publications Office of the European Union, Luxembourg, 2019).

13. UNPD. *World Population Prospects 2019, Online Edition. Rev. 1.* (United Nations, Department of Economic and Social Affairs, Population Division, 2019).

14. Liu, Z. *et al*. Reduced carbon emission estimates from fossil fuel combustion and cement production in China. *Nature* **524**, 335-8 (2015).

15. Shan, Y. *et al*. New provincial CO_2 emission inventories in China based on apparent energy consumption data and updated emission factors. *Appl. Energy* **184**, 742-750 (2016).

16. Shan, Y., Guan, D., Zheng, H., Ou, J. & Qiang, Z. China CO_2 emission accounts 1997–2015. *Sci. Data* **5**, 170201 (2018).

17. Algunaibet, I. M. *et al*. Correction: powering sustainable development within planetary boundaries. *Energy & Environmental Science* **12**, 3612 (2019).

18. Rockström, J. *et al*. Planetary boundaries: exploring the safe operating space for humanity. *Ecol. Soc.* **14**, 292-292 (2009b).

19. Raworth, K. A Safe and Just Space for Humanity: Can We Live Within the Doughnut? (Oxfam, Oxford, UK, 2012).

20. Tian, W. M. Calculation of Provincial Gini coefficient and analysis on the trends. *Economic Science* **2**, 48–59 (2015).

21. Shi, T., Yang, S., Zhang, W. & Zhou, Q. Coupling coordination degree measurement and spatiotemporal heterogeneity between economic development and ecological environment ----Empirical evidence from tropical and subtropical regions of China. *J. Clean Prod.* **244**, 118739 (2020).

22. Li, L., Fan, Z., Feng, W., Yuxin, C. & Keyu, Q. Coupling coordination degree spatial analysis and driving factor between socio-economic and eco-environment in northern China. *Ecol. Indic.* **135**, 108555 (2022).

23. Bryan, B. A., Gao, L., Ye, Y., Sun, X. & Hou, X. China's response to a national land-system sustainability emergency. *Nature* **559**, 193-204 (2018).

24. Folke, C., Carpenter, S. R., Walker, B., Scheffer, M., Chapin, T. & Rockström, J. Resilience thinking: integrating resilience, adaptability and transformability. *Ecol. Soc.* **15**, 299-305 (2010).

25. Biggs, R., Schlüter, M. & Schoon, M. L. An Introduction to the Resilience Approach and Principles to Sustain Ecosystem Services in Social-Ecological Systems. In: *Principles for Building Resilience* (Cambridge: Cambridge University Press, 2015).

26. Wu, J. Landscape sustainability science: ecosystem services and human well-being in changing landscapes. *Landsc. Ecol.* **28**, 999-1023 (2013).

27. Atmospheric Composition Analysis Group. *Surface $PM_{2.5}$*. https://sites.wustl.edu/acag/datasets/surface-pm2-5/#V5.GL.02 (2021).

28. van Donkelaar, A. *et al*. Monthly Global Estimates of Fine Particulate Matter and Their Uncertainty. *Environmental Science & Technology* **55**, 15287-15300 (2021).

29. Ministry of Ecology and Environment of the People's Republic of China. *Ambient air quality standards GB 3095–2012*. (China Environmental Science Press, Beijing, 2012).

30. World Health Organization. *WHO Air quality guidelines for particulate matter, ozone, nitrogen dioxide and sulfur dioxide*. (WHO, Geneva, 2005).

31. World Health Organization. *WHO global air quality guidelines: particulate matter ($PM_{2.5}$ and PM_{10}), ozone, nitrogen dioxide, sulfur dioxide and carbon monoxide*. (WHO, Geneva, 2021).

32. Cheng, J. *et al*. Pathways of China's $PM_{2.5}$ air quality 2015–2060 in the context of carbon neutrality. *Natl. Sci. Rev.* **12**, 12 (2021).

33. Zhang, Q., Zheng, Y., Tong, D., Shao, M. & Hao, J. Drivers of improved $PM_{2.5}$ air quality in China from 2013 to 2017. *Proc. Natl. Acad. Sci.* **116**, 201907956 (2019).

34. Zheng, B. *et al*. Trends in China's anthropogenic emissions since 2010 as the consequence of clean air actions. *Atmospheric Chemistry & Physics Discussions* **18**, 14095-14111 (2018).

35. Sandalow, D. *Guide to Chinese Climate Policy 2019*. https://energypolicy.columbia.edu/research/report/guide-chinese-climate-policy (2019).

36. Qiu, J. *et al*. Understanding relationships among ecosystem services across spatial scales and over time. *Environ. Res. Lett.* **13**, 054020 (2018).
