# Supplementary material for: Assessing coupling interactions in a safe and just operating space for regional sustainability
Source: Nat Commun. 2023 Mar 13;14:1369. doi: 10.1038/s41467-023-37073-z (PMC10011604; doi:10.1038/s41467-023-37073-z)
Supplement: Supplementary file 3 — Reporting Summary [file 41467_2023_37073_MOESM3_ESM.pdf]

nature portfolio

Corresponding author(s): Deyong Yu

Last updated by author(s): Jan 22, 2023

Reporting Summary

Nature Portfolio wishes to improve the reproducibility of the work that we publish. This form provides structure for consistency and transparency in reporting. For further information on Nature Portfolio policies, see our Editorial Policies and the Editorial Policy Checklist.

Statistics

For all statistical analyses, confirm that the following items are present in the figure legend, table legend, main text, or Methods section.

n/a	Confirmed	
☒	☐	The exact sample size (*n*) for each experimental group/condition, given as a discrete number and unit of measurement
☒	☐	A statement on whether measurements were taken from distinct samples or whether the same sample was measured repeatedly
☐	☒	The statistical test(s) used AND whether they are one- or two-sided<br>*Only common tests should be described solely by name; describe more complex techniques in the Methods section.*
☒	☐	A description of all covariates tested
☒	☐	A description of any assumptions or corrections, such as tests of normality and adjustment for multiple comparisons
☐	☒	A full description of the statistical parameters including central tendency (e.g. means) or other basic estimates (e.g. regression coefficient) AND variation (e.g. standard deviation) or associated estimates of uncertainty (e.g. confidence intervals)
☒	☐	For null hypothesis testing, the test statistic (e.g. *F*, *t*, *r*) with confidence intervals, effect sizes, degrees of freedom and *P* value noted<br>*Give P values as exact values whenever suitable.*
☒	☐	For Bayesian analysis, information on the choice of priors and Markov chain Monte Carlo settings
☒	☐	For hierarchical and complex designs, identification of the appropriate level for tests and full reporting of outcomes
☒	☐	Estimates of effect sizes (e.g. Cohen's *d*, Pearson's *r*), indicating how they were calculated

Our web collection on statistics for biologists contains articles on many of the points above.

Software and code

Policy information about availability of computer code

Data collection	No code or software was used in data collection.
Data analysis	Data processing in this study was conducted at ArcGIS 10.2, Microsoft 2016, Origin 2018, and R (version 3.4.3). In the analysis in R, ggplot2 and gbm packages were used.

For manuscripts utilizing custom algorithms or software that are central to the research but not yet described in published literature, software must be made available to editors and reviewers. We strongly encourage code deposition in a community repository (e.g. GitHub). See the Nature Portfolio guidelines for submitting code & software for further information.

Data

Policy information about availability of data

All manuscripts must include a data availability statement. This statement should provide the following information, where applicable:

- Accession codes, unique identifiers, or web links for publicly available datasets
- A description of any restrictions on data availability
- For clinical datasets or third party data, please ensure that the statement adheres to our policy

The global data used for comparison are mainly from EDGAR (http://edgar.jrc.ec.europa.eu/), FAOSTAT (http://faostat.fao.org/), World Bank (https://data.worldbank.org/) and Eora MRIO (http://worldmrio.com/) databases. World population is from UNPD database (https://population.un.org/wpp/). China's provincial data are from CEADS database (www.ceads.net/data/), Resource and Environment Science and Data Center (https://www.resdc.cn/) and China Statistical

Yearbooks. Additional environmental data for China are from ACAG (https://sites.wustl.edu/acag/datasets/surface-pm2-5/#V5.GL.02) database and China Statistical Yearbooks. All the data generated in this study are provided in the Supplementary Information/Source Data file.

Human research participants

Policy information about studies involving human research participants and Sex and Gender in Research.

Reporting on sex and gender	Our findings do not applied to only one sex or gender. In our research, we studied gender equality in Sustainable Development Goals. The source data of gender equality are obtained from Word Bank database and China Statistical Yearbooks.
Population characteristics	See above
Recruitment	No sampling in this study, we have studied all population in China.
Ethics oversight	No sampling in this study, we have studied all population in China.

Note that full information on the approval of the study protocol must also be provided in the manuscript.

Field-specific reporting

Please select the one below that is the best fit for your research. If you are not sure, read the appropriate sections before making your selection.

☐ Life sciences ☐ Behavioural & social sciences ☒ Ecological, evolutionary & environmental sciences

For a reference copy of the document with all sections, see nature.com/documents/nr-reporting-summary-flat.pdf

Ecological, evolutionary & environmental sciences study design

All studies must disclose on these points even when the disclosure is negative.

Study description	We downscaled four planetary boundaries (climate change, freshwater use, land-system change, biogeochemical flows), estimated corresponding environmental footprints (carbon footprint, blue water footprint, land footprint, and nitrogen & phosphorus footprint), and measured safe and just operating space in China from 2000 to 2018. Further, we assessed China's (un)sustainability performance and trends relative to safe and just operating space and Sustainable Development Goals, and interactions between environmental performance and human well-being using coupling coordination degree (CCD) model. In addition, we further delineated four development patterns (i.e., coupled and developed, coupled and underdeveloped, uncoupled and underdeveloped, and coupled and underdeveloped) on the level of coupling (i.e., magnitude of CCD) and development (i.e., CCD trend), and developed targeted strategies and pathways for regions to transition towards sustainability.
Research sample	This study is a nation-wide research, all regions are included in this study. We did not do a specific sampling. However, the data for Tibet, Taiwan, Hongkong, and Macao were not available, we studied 30 provinces in China.
Sampling strategy	We did not do a specific sampling, because all provinces are included in the study. However, the data for Tibet, Taiwan, Hongkong, and Macao were not available, we studied 30 provinces in China.
Data collection	We downloaded all the data from publicly available databases on the Internet, collected in March-May of 2020.
Timing and spatial scale	Due to the limits of data availability, we performed long time series whenever possible. We study China at national, regional, and provincial scales from 2000 to 2018.
Data exclusions	No data were excluded in this study.
Reproducibility	Data were processed 3 times at different time intervals leading to the same results.
Randomization	NA, we have used all provinces in China with no sampling.
Blinding	NA, we have used all provinces with no sampling.

Did the study involve field work? ☐ Yes ☒ No

Reporting for specific materials, systems and methods

We require information from authors about some types of materials, experimental systems and methods used in many studies. Here, indicate whether each material, system or method listed is relevant to your study. If you are not sure if a list item applies to your research, read the appropriate section before selecting a response.

Materials & experimental systems

n/a	Involved in the study
☒	☐ Antibodies
☒	☐ Eukaryotic cell lines
☒	☐ Palaeontology and archaeology
☒	☐ Animals and other organisms
☒	☐ Clinical data
☒	☐ Dual use research of concern

Methods

n/a	Involved in the study
☒	☐ ChIP-seq
☒	☐ Flow cytometry
☒	☐ MRI-based neuroimaging
